# The endophytic *Fusarium* strains: a treasure trove of natural products

**DOI:** 10.1039/d2ra04126j

**Published:** 2023-01-09

**Authors:** Arwa Mortada Ahmed, Basma Khalaf Mahmoud, Natalie Millán-Aguiñaga, Usama Ramadan Abdelmohsen, Mostafa Ahmed Fouad

**Affiliations:** a Department of Pharmacognosy, Faculty of Pharmacy, Deraya University 61111 New Minia City Egypt; b Department of Pharmacognosy, Faculty of Pharmacy, Minia University 61519 Minia Egypt usama.ramadan@mu.edu.eg +20-86-2369075 +20-86-2347759; c Universidad Autónoma de Baja California, Facultad de Ciencias Marinas Carretera Transpeninsular Ensenada-Tijuana No. 3917, Colonia Playitas Ensenada Baja California 22860 Mexico

## Abstract

The complexity and structural diversity of the secondary metabolites produced by endophytes make them an attractive source of natural products with novel structures that can help in treating life-changing diseases. The genus *Fusarium* is one of the most abundant endophytic fungal genera, comprising about 70 species characterized by extraordinary discrepancy in terms of genetics and ability to grow on a wide range of substrates, affecting not only their biology and interaction with their surrounding organisms, but also their secondary metabolism. Members of the genus *Fusarium* are a source of secondary metabolites with structural and chemical diversity and reported to exhibit diverse pharmacological activities. This comprehensive review focuses on the secondary metabolites isolated from different endophytic *Fusarium* species along with their various biological activities, reported in the period from April 1999 to April 2022.

## Abstract 2

The complexity and structural diversity of the secondary metabolites produced by endophytes make them an attractive source of natural products with novel structures that can help in treating life-changing diseases.

## Graphical abstract

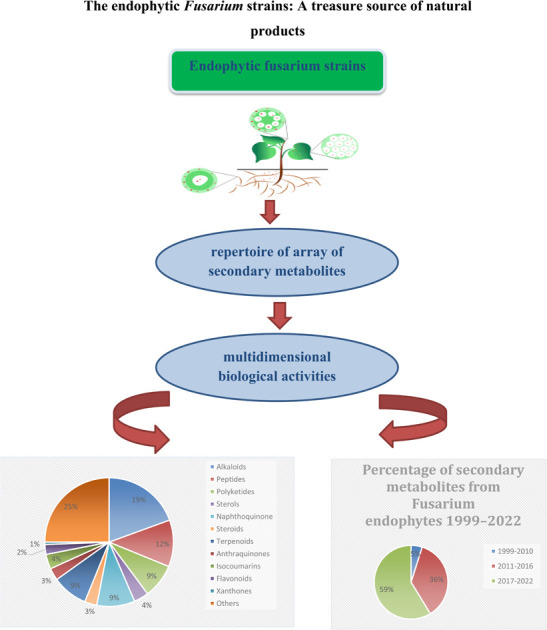

## 1. Introduction

Currently, there is an urgent need for the discovery of new molecules to overcome the challenges that threaten human life. Health-related problems such as antimicrobial resistance, life-threating viruses such as Covid-19, SARS, bird flu, and AIDS, and cancer seriously affect people's health.^1^ Scientists have been fascinated by finding secondary metabolites with novel skeletons from new natural sources.^2^ One of the great sources of novel natural products for utilization in medicine and other fields is the entophytic microorganisms, which survive internally in living tissues and are ubiquitous in all medicinal plant species.^3^ Endophytic microorganisms (fungi or bacteria) are capable of producing the same or similar compounds to those of the host plant without causing harm or apparent disease.^4^ Endophytes provide benefits in survival, biodiversity, and ecosystem abilities to enhance the response to environmental stress.^4^ Endophytes might be involved in the biosynthesis of plant products; however, they might also be the producers themselves of many substances of potential use to modern medicine, agriculture and the pharmaceutical industry.^5^

**Fig. 1:**
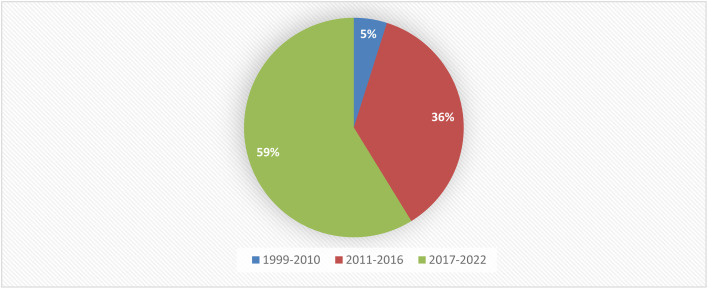
Percentage of secondary metabolites produced by *Fusarium* genus between 1999 and 2022.

Fungal endophytes are a major source of anti-infective agents and other medically relevant compounds.^6^ Endophytic fungi are a source of a diverse array of multidimensional bioactive secondary metabolites such as alkaloids, terpenoids, steroids, quinones, iso-coumarins, lignans, phenylpropanoids, phenols, and lactones.^7^ In 2013, the Food and Drug Administration (FDA) reported that 25% of drugs were discovered from micro-organisms, among 38% natural products that were used as drugs.^8^*Fusarium* is considered as a member of the most dominant endophytic fungal genera in the world, characterized genetically with extraordinary discrepancy, together with its ability to grow on a wide range of substrates and their efficient mechanisms for dispersal, which affect their biology and interaction with their surrounding organisms, together with secondary metabolism that makes *Fusarium* an important group of fungi.^8–10^*Fusarium* genus is in the third level of endophytes after *Aspergillus* and *Penicillium* sp. and it is a cosmopolitan genus of filamentous ascomycete fungi (*Sordariomycetes*, *Hypocreales*, *Nectriaceae*), which comprise more than 70 species with a wide range of hosts.^8–10^ They are widely abundant in soil, subterranean and aerial plant parts, plant debris, and other organic substrates.^11^*Fusarium* is considered to be a rich source of bioactive compounds, including more than one hundred compounds with unique chemical structures, among more than three hundred compounds of various classes such as butenolides, alkaloids, terpenoids, cytochalasins, phenalenones, xanthones, sterols, and diphenyl ether and anthraquinone derivatives, with multidimensional bioactivities such as antimicrobial, antiviral, anticancer, antioxidant, antiparasitic and immunomodulating activity.^8^ There are many examples of fungal endophytes that have been reported to produce bioactive metabolites similar to those originally derived from the host plant.^12^ The majority of *Fusarium* metabolites were isolated in the period from 2017 to 2022, see Fig. 1. This review covers the literature on the isolated natural products from the fungal endophyte *Fusarium* species and their various bioactivities from April 1999 to April 2022.

## 2. Phylogenetic analysis

**Fig. 2:**
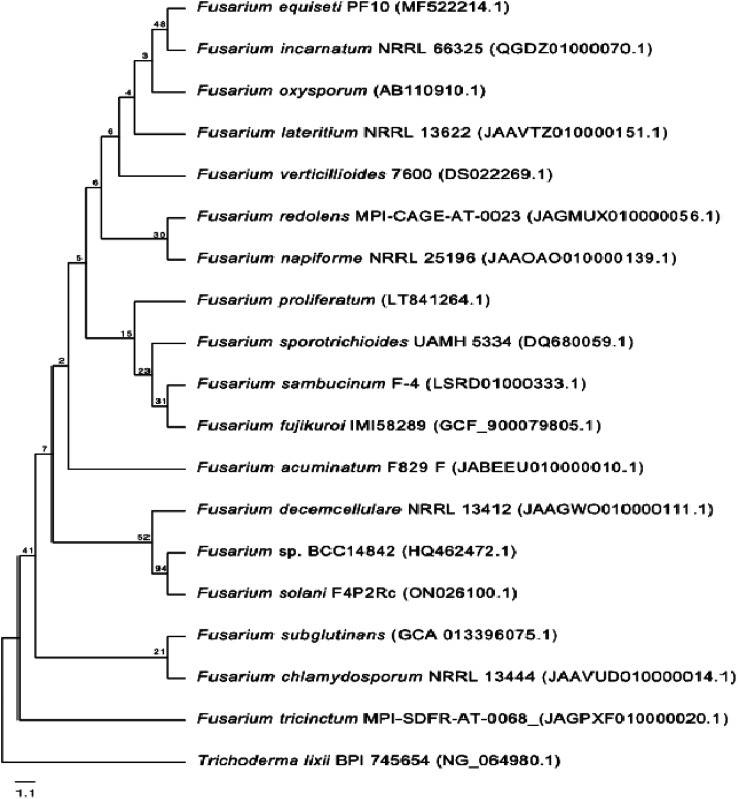
Neighbour-joining tree based on 18S rRNA gene sequences (∼1037 bp length) of different *Fusarium* species. The *Trichoderma* species was used as an outgroup. The tree includes accession numbers in parentheses and bootstrap values.

One phylogenetic tree was established according to the ribosomal 18S RNA gene sequence analysis, as shown in Fig. 2. The genus *Trichoderma* was used as an outgroup to gain a better overview of the diversity of *Fusarium* species. Even though the 18S rRNA gene is highly conserved, the *Fusarium* species clustered into different clades, confirming the high diversity of this genus and the diversity of chemical compounds produced by the different species of *Fusarium*, as mentioned in this review. Nucleotide sequences of 18S rRNA of *Fusarium* species and the outgroup *Trichoderma* were extracted from the National Center for Biotechnology Information (NCBI, https://www.ncbi.nlm.nih.gov/). The 18S rRNA sequences were aligned using Muscle (maximum number of iterations of 10) implemented in Geneious Prime 2022.1.1 (https://www.geneious.com). The alignment was manually curated and trimmed, and the best nucleotide model (Kimura 2-parameter + gamma distribution) was determined using MEGA X.^13^ A neighbour-joining tree was computed using MEGA X with 1000 bootstrap replicates and the best model. It was shown that all of the different members of *Fusarium* species could be grouped together in a single cluster relative to the outgroup *Trichoderma* species.

### 2.1. F. chlamydosporum

The endophytic fungus *F. chlamydosporum* has been isolated from different plants, such as the *Suaeda glauca* root, and is a source of various pharmacologically active secondary metabolites including: three indole derivatives (1–3) identified as chlamydosporin (1), methyl indol-3-ylacetate (2), and Nb-acetyltryptamine (3), three cyclohexadepsipeptides known as destruxin A4 (4), trichomide B (5), and homodestcardin (6) and four pyrones (7–10) known as kojic acid (7), kojic acid monomethyl ether (8), 5-hydroxy-4-oxo-4*H*-pyran-2-ethyl formate (9) and phomapyrone C (10).^14^ Compounds (1) and (2) exhibited significant phytotoxic activity against the radicle growth of *Echinochloa crusgalli* and *Amaranthus retroflexus*, wherein the first showed inhibition rates greater than 80 and 77.0%, in 10 μg mL^−1^ solutions, respectively, while the latter showed moderate activity with inhibition rates lower than 40%, better than the positive control (2,4-dichlorophenoxyacetic acid, 2,4-D).^14^ Cyclohexadepsipeptides (4–6) and pyrones (7–10) showed various brine shrimp lethality, especially destruxin A4 (4) and kojic acid (7) with significant LD_50_ values of 2.78 and 7.40 μg mL^−1^, which are better than the positive control colchicine (at 7.75 μg mL^−1^). Destruxin A4 (4) induced 5-fold erythropoietin gene expression at a concentration of 0.2–2 μM; erythropoietin (EPO) is a primary hormone that regulates the proliferation and differentiation of immature erythroid cells.^15^ On the other hand, compounds 1, 3, 9, and 10 showed weak antimicrobial activity against different pathogens such as *Staphylococcus aureus*, *Escherichia coli*, and *Salmonella enterica*, as well as the two plant pathogenic fungi *Rhizoctonia cerealis* and *Colletotrichum gloeosporioides* with MIC values of 62.5–250 μg mL^−1^.^14^ Furthermore, *F. chlamydosporum*, isolated from *Anvillea garcinii* (Asteraceae) leaves, was found to be a source of two new ergosterol derivatives, namely chlamydosterols A (11) and B (12), together with three known ergosterols: ergosta-7,22-dien-3β-ol (13), ergosta-5,7,22-triene-3β-ol (14) and ergosta-7,22-diene-3β,5α,6*b*-triol (15).^16^

**Fig. 3:**
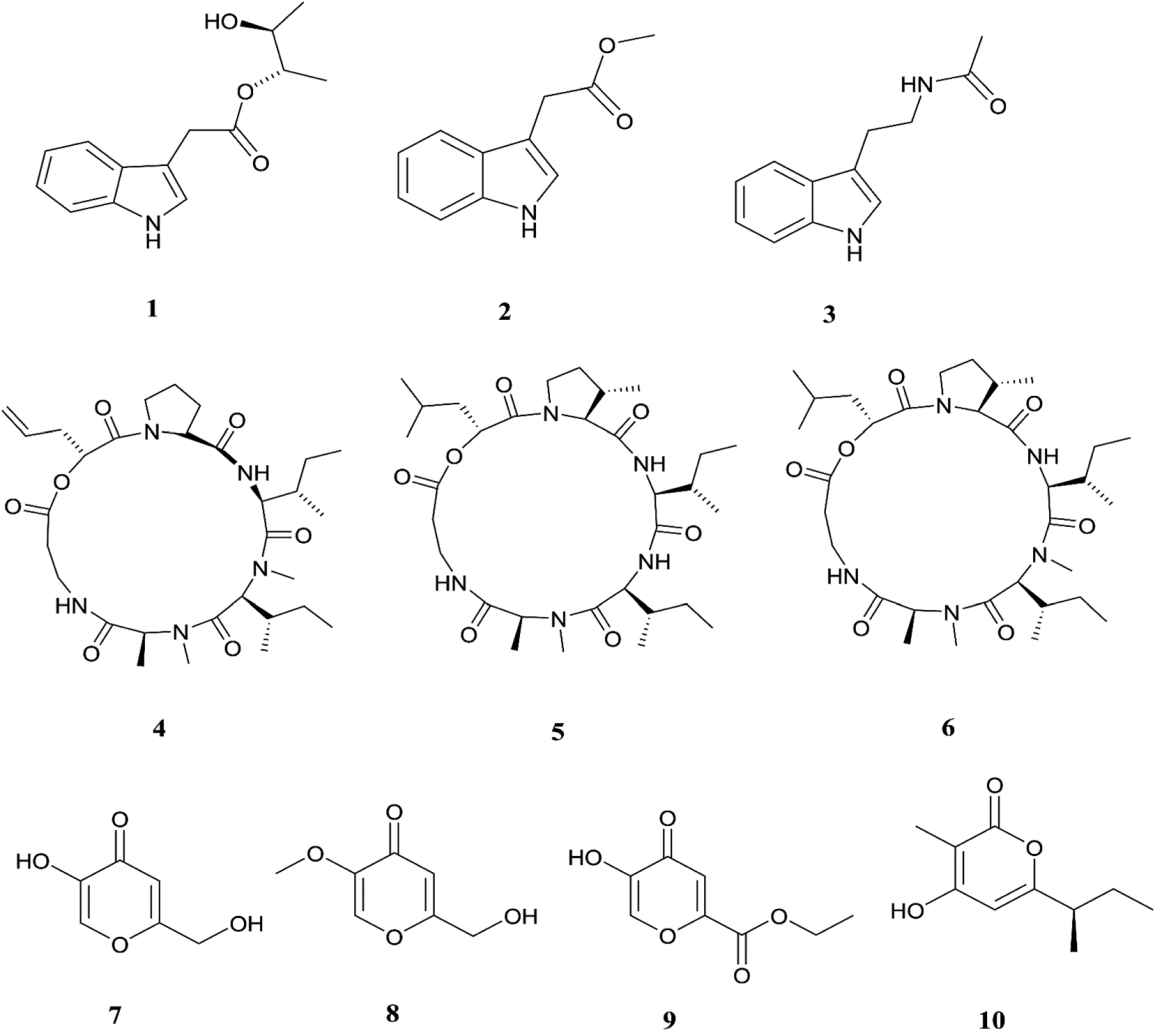
Various biologically active compounds (1–10) isolated from *F. chlamydosporum*.

**Fig. 4:**
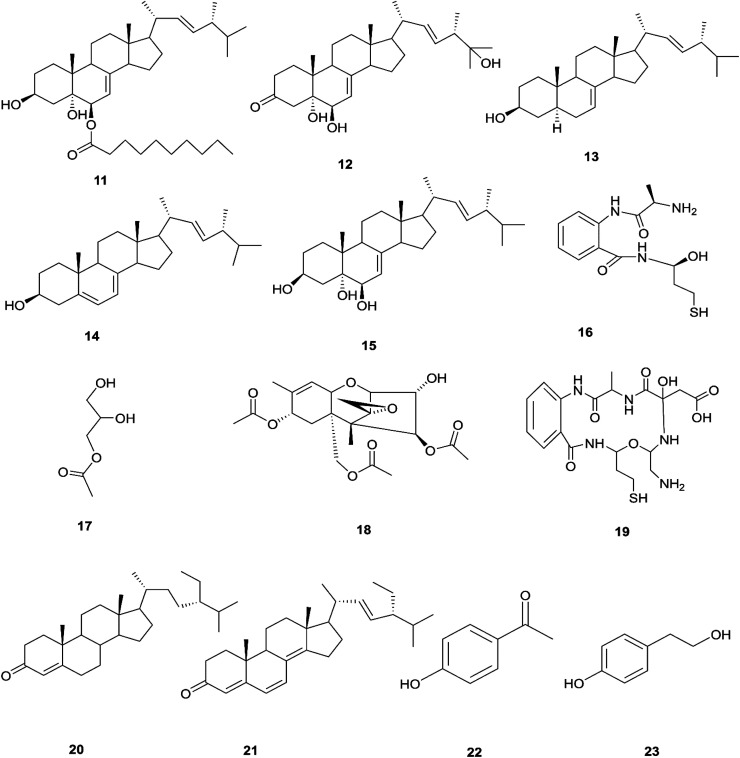
Various biologically active compounds (11–23) isolated from *F. chlamydosporum*.

It was reported that ergosterol and an ergosterol-enriched fraction prohibited the production of tumour necrosis factor-α (TNF-α), nitric oxide (NO), and nuclear factor kappa-light-chain enhancer of activated B cell luciferase activity in macrophage RAW 264.7 cells.^17^ Only chlamydosterol A (11) and ergosta-5,7,22-triene-3β-ol (14) possess anti-inflammatory activity with moderate inhibition activity against the 5-lipoxygenase (5-LOX) enzyme with IC_50_ values of 3.06 and 3.57 μM, respectively, while (12), (13), and (15), exhibited weak activity with IC_50_ values of 7.23, 4.18, and 5.46 μM, compared to indomethacin (IC_50_: 1.13 μM).^16^ Additionally, a new benzamide derivative, fusarithioamide A (16), along with 1-*O*-acetyl-glycerol (17) and 8-acetylneosolaniol (18), was isolated from the ethyl acetate extract of the same strain, and is a good new candidate as a potential antimicrobial and cytotoxic agent as it displayed potent and selective cytotoxic activity toward BT-549 and SKOV-3 cell lines with IC_50_ values of 0.4 and 0.8 μM, respectively, compared to doxorubicin (IC_50_ 0.046 and 0.313 μM, respectively). Fusarithioamide A also possessed potent antibacterial activity with MIC values of 3.1, 4.4, and 6.9 mg mL^−1^ and inhibition zone diameters (IZDs) of 19.0, 14.1, and 22.7 mm against *B. cereus*, *S. aureus*, and *E. coli*, respectively, compared to ciprofloxacin (IZDs of 20.7, 16.6, and 26.9 mm, respectively). Further extensive investigation of this extract resulted in the identification of a new aminobenzamide derivative, fusarithioamide B (19), and four known metabolites: stigmast-4-ene-3-one (20), stigmasta-4,6,8(14),22-tetraen-3-one (21), *p*-hydroxyacetophenone (22) and tyrosol (23).^18^ Furthermore, fusarithioamide B displayed selective cytotoxic potency toward the BT-549, MCF-7, SKOV-3, and HCT-116 cell lines with IC_50_ values of 0.09, 0.21, 1.23, and 0.59 μM, compared to doxorubicin (IC_50_ values of 0.046, 0.05, 0.321, and 0.24 μM, respectively); it also showed selective antifungal activity toward *C. albicans* (MIC of 1.9 μg mL^−1^ and IZD of 14.5 mm), compared to clotrimazole (MIC of 2.8 μg mL^−1^ and IZD of 17.9 mm); finally, it also possessed potent antibacterial potential toward *E. coli* (IZD of 25.1 mm; MIC of 3.7 mg mL^−1^), *B. cereus* (IZD of 23.0 mm; MIC of 2.5 mg mL^−1^), and *S. aureus* (IZD of 17.4 mm; MIC of 3.1 mg mL^−1^) compared to ciprofloxacin (IZD/MIC: 25.6/3.9, 21.2/2.9, and 15.3/3.4 mm mg^−1^ mL^−1^, respectively). It was also reported that the antimicrobial effect of sulfur compounds could be attributed to their reaction with sulfhydryl moieties of the microorganism cellular protein to disturb the cellular metabolism, therefore fusarithioamide B is considered a future developing lead molecule for antitumor and antimicrobial agents.^18^ Tyrosol (23) is a natural phenylethanoid that has been extracted from olive oil and shows anti-inflammatory activity due to its satisfactory binding affinity with COX-2 (PDB ID: 5F1A) as compared to other NSAIDs (Aspirin, Ibuprofen, and Naproxen) and a COX-2 selective drug (Celecoxib).^19^ The chemical structures of 1 to 23 are shown in Fig. 3 and 4.

### 2.2. F. solani

*F. solani* is one of the most important sources of novel and diverse pharmacologically active secondary constituents. *F. solani* is isolated from *Taxus Brevifolia*, which is considered to be an additional source of Taxol (24) that is the first member of the taxane family to be used in cancer chemotherapy and is characterized by its limited availability, high cost and low yield from plant sources; it is used in the treatment of several different types of cancer, such as breast, ovarian, prostate, non-small cell lung, adeno-carcinoma, and esophagus squamous cell carcinoma. It inhibited cell proliferation of a number of cancer cell lines such as HeLa, HepG2, Jurkat, Ovcar3 and T47D with IC_50_ values ranging from 0.005 to 0.2 μM for fungal taxol.^20^ It induced apoptosis in JR4-Jurkat cells with a possible involvement of anti-apoptotic Bcl_2_ and loss in mitochondrial membrane potential and was unaffected by inhibitors of caspase-9, -2 or -3 but was prevented in the presence of caspase-10 inhibitor.^20^ However, significant toxicities, such as myelosuppression and peripheral neuropathy, limit the effectiveness of paclitaxel-based treatment regimens.^21^*F. solani* was the only one strain among 153 endophytic fungi recovered from the roots, stems, and leaves of *Cajanus cajan* that was found to produce vitexin (5,7,4-trihydroxyflavone-8-glucoside) (25), which is well known to have valuable biological properties such as anti-inflammatory, anticancer, anti-nociceptive, antioxidant, anti-convulsant, cardioprotective, hypotensive, memory enhancing potential, and anti-diabetic.^22^

Recently, there was a study that revealed the good osteogenic proliferation stimulating activity of vitexin that made it a lead molecule for the treatment of osteoporosis^22^ The anti-neoplastic effects are due to the promotion of apoptosis and autophagy as well as the inhibition of proliferation and migration through several signalling pathways.^23^ Vitexin exhibited an anti-hyperalgesic effect through reducing the pro-inflammatory cytokine (TNF-α, IL-1β, IL-6, and IL-33) and enhancing the anti-inflammatory cytokine (IL-10) production induced by carrageenan.^23^ Vitexin exhibits a protective effect against cardiac ischemia/reperfusion (I/R) injury through inhibiting the I/R-induced decrease in coronary flow and ST segment elevation in EKG.^23^ Vitexin significantly reduced postprandial blood glucose both in sucrose loaded normoglycemic mice and sucrose induced diabetic rats, which demonstrates a potential role on diabetes.^23^ Additionally, seven secondary metabolites were isolated from *F. solani* (*Cassia alata* Linn root), including: three naphthaquinones, anhydrofusarubin (26), fusarubin (27) and 3-deoxyfusarubin (28), one aza-anthraquinone, bostrycoidin (29), two sterols, ergosterol (30) and 3,5,9-trihydroxyergosta-7,22-diene-6-one (31), and 4-hydroxybenzaldehyde (32).^24^ Interestingly, fusarubin (27) exhibited a significant neuroprotective activity on glutamate-mediated HT22 cell death; fusarubin has been known as an inhibitor of mitochondrial NADH ubiquinone reductase, which is also known as coenzyme Q10 and is well known as a free-radical-scavenging antioxidant.^25^ These studies confirm the hypothesis that endophytes can be used for the industrial production of important neuroprotective drugs. Likewise, these compounds displayed varied cytotoxic potency on vero cells, wherein 4-hydroxybenzaldehyde (32) and bostrycoidin (29) cause the death of nearly 25% of the cells, while anhydrofusarubin (26) and 3,5,9-trihydroxyergosta-7,22-diene-6-one(31) killed 35% of the cells, indicating that the cell proliferation inhibition activity of these four compounds would be beneficial in the anticancer treatment of kidney cancer patients.^24^ In addition, fusarubin (27) exhibited highly significant antibacterial activity against four pathogens, *B. megaterium*, *S. aureus*, *P. aeruginosa* and *E. coli*, with inhibition zones of 21–32 mm, while bostrycoidin (29) and anhydrofusarubin (26) displayed prominent potency with inhibition zones of 12–16 mm and 10–17 mm, respectively.^24^

**Fig. 5:**
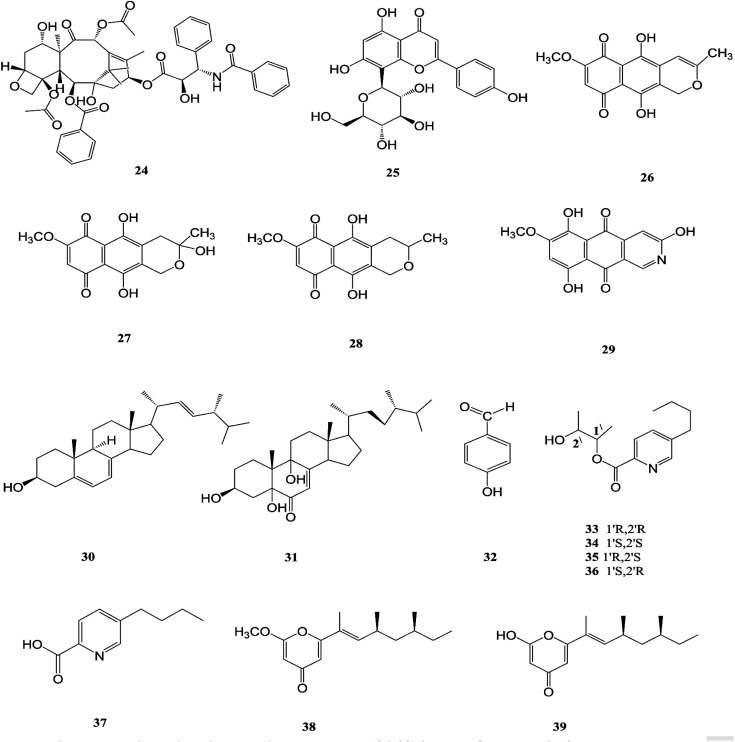
Various biologically active compounds (24–39) isolated from *F. solani*.

**Fig. 6:**
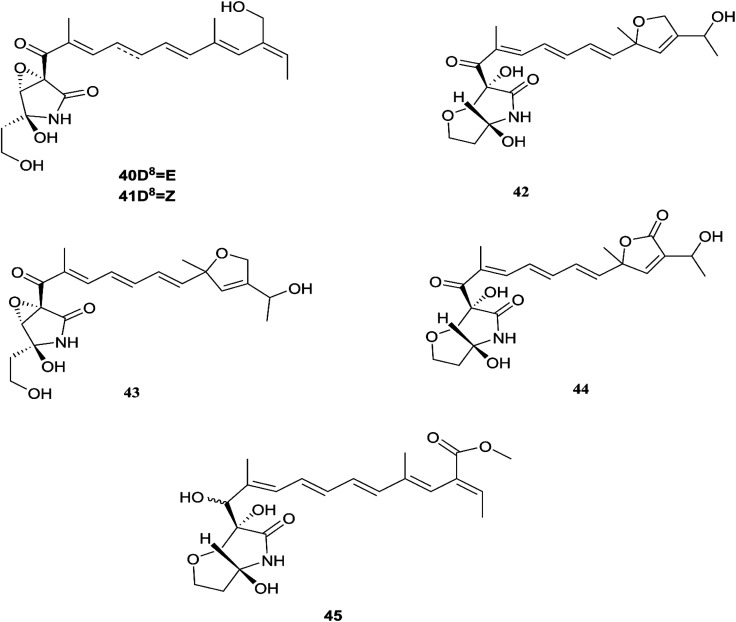
Various biologically active compounds (40–45) isolated from *F. solani*.

**Fig. 7:**
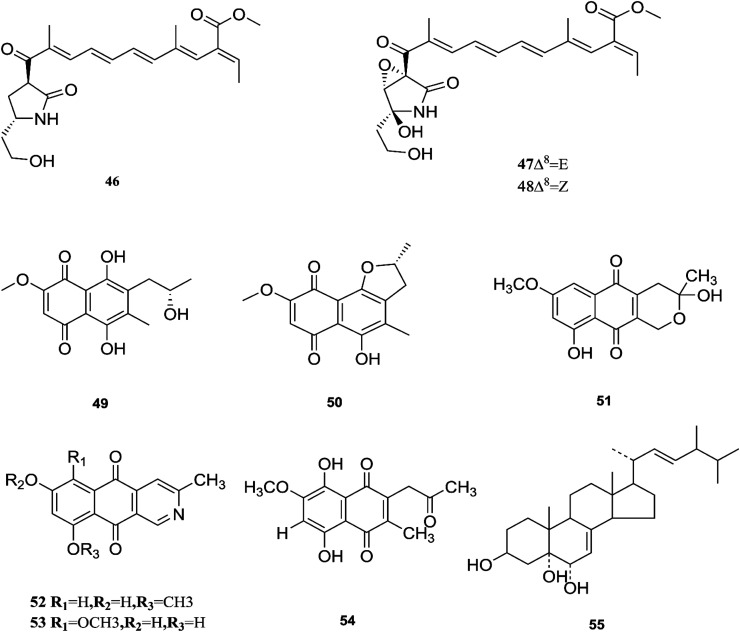
Various biologically active compounds (46–55) isolated from *F. solani*.

Fusarubin (27) and anhydrofusarubin (26) inhibit proliferation and increase apoptosis in cell lines derived from hematological cancers like acute myeloid leukemia (OCI-AML3) and other hematological tumor cell lines (HL-60, U937, and Jurkat); fusarubin was more potent than anhydrofusarubin, due to up-regulating p_21_ expression in a p_53_-dependent manner resulting in decreasing ERK phosphorylation and increasing p_38_ expression, both of which increase p_21_ stability.^26^ The antimicrobial activity of bostricoidin and fusarubin toward *Pseudomonas aeruginosa* is due to stimulation of respiratory oxidation of bacterial cells and inducing cyanide-insensitive oxygen consumption.^27^ Finally, bostrycoidin, anhydrofusarubin, 4-hydroxybenzaldehyde and fusarubin showed significant free radical scavenging activity with IC_50_ values of 1.6 12.4, 28.9 and 34.8 μg mL^−1^, respectively, compared to BHA, trolox and ascorbic acid as positive controls (IC_50_ values of 1.2, 1.3 and 1.5 μg mL^−1^, respectively).^24^ Investigation of *F. solani* HDN15-410, which is a mangrove-derived fungus, isolated from the root of *Rhizophora apiculata* Blume, resulted in the isolation of five fusaric acid derivatives, fusaricate H–K (33–36) and fusaric acid (37), as well as two undescribed γ-pyrone derivatives, fusolanone A (38) and B (39), see Fig. 5. Fusolanone B (39) demonstrated the best antimicrobial activity against *Vibrio parahaemolyticus* with a MIC value of 6.25 μg mL^−1^.^28^ Fusaric acid (37) has a potent cytotoxic effect on the HeLa cell line with an IC_50_ value of 200 μg mL^−1^ at 24 h.^29^ Furthermore, the chemical investigation of *F. solani* JK10, which is harbored in the root of the Ghanaian medicinal plant *Chlorophora regia*, resulted in the isolation of seven new 7-desmethyl fusarin C derivatives (40–46) together with five known compounds, NG-391(47), NG-393 (48), (+)-(*S*)-solaniol (49), 2,3-dihydro-5-hydroxy-8-methoxy-2,4-dimethylnaphtho[1,2-*b*]furan-6,9-dione (50) and Nb–acetyltryptamine (3). Consequently, compounds 42/43 and 45 showed pronounced activity at 10.0 μg mL^−1^ against the soil bacterium *Acinetobacter* sp. BD4 comparable to streptomycin as a reference standard at 10.0 μg mL^−1^. Additionally, 44 and 46 revealed high activity against *E. coli* at 5.0 μg mL^−1^.^30^ Their chemical structures are shown in Fig. 6 and 7.

One new naphthoquinone, 9-desmethylherbarine (51), and two aza-anthraquinone derivatives, 7-desmethylscorpinone (52) and 7-desmethyl-6-methylbostrycoidin (53), along with four known compounds, anhydrofusarubin (26), fusarubin (27), javanicin (54) and cerevesterol (55), were reported for the first time in the *Fusarium* genus that was isolated from *F. solani* (*Aponogeton undulates*). In addition, compounds 52 and 53 exerted cytotoxic activity against four human tumor cell lines, *i.e.*, HeLa cervical carcinoma, MDA MB 231 breast cancer, MIA PaCa2 pancreatic cancer, and NCI H1975 non-small-cell lung cancer, with low micromolar to submicromolar IC_50_ values of 0.96 μM, 1.51 μM, 0.98 μM, and 0.61 μM, respectively, for compound 52, and 0.71, 0.73, 0.64, and 0.34 μM, respectively, for compound 53, while compound 51 was weakly active with values of 10.3, 30.72, 20.46, and 27.73 μM, respectively, using doxorubicin as a positive control (0.05, 0.07, 0.04, and 0.03 μM).^31^ Additionally, two new polyhydroxylated steroids, 2β,9α-dihydroxy-5α-methoxyergosta-7,22-diene (56) and 2β,6β-dihydroxy-5α-methoxyergosta-7,22-diene (57), were isolated from *F. solani* found in *Chloranthus multistachys* leaves and showed antagonistic properties against seven pathogens (*Glomerella cingnlata*, *Alternaria brassicae*, *Penicillium digitatum*, *Botrytis cinerea*, *Verticillium alboatrum*, *Phytophthora capsici* and *Exserohilum turcicum*) with GI ≥ 70%, proving their broad spectrum antimicrobial properties.^32^ Camptothecin (58) and 10-hydroxycamptothecin (59) are considered to be very important precursors for the clinically potent anticancer drugs topotecan and irinotecan, and 9-methoxycamptothecin (60) was isolated from the endophytic fungal strains in *Camptotheca acuminata* and also from *Apodytes dimidiata* (*Icacinaceae*).^33^

**Fig. 8:**
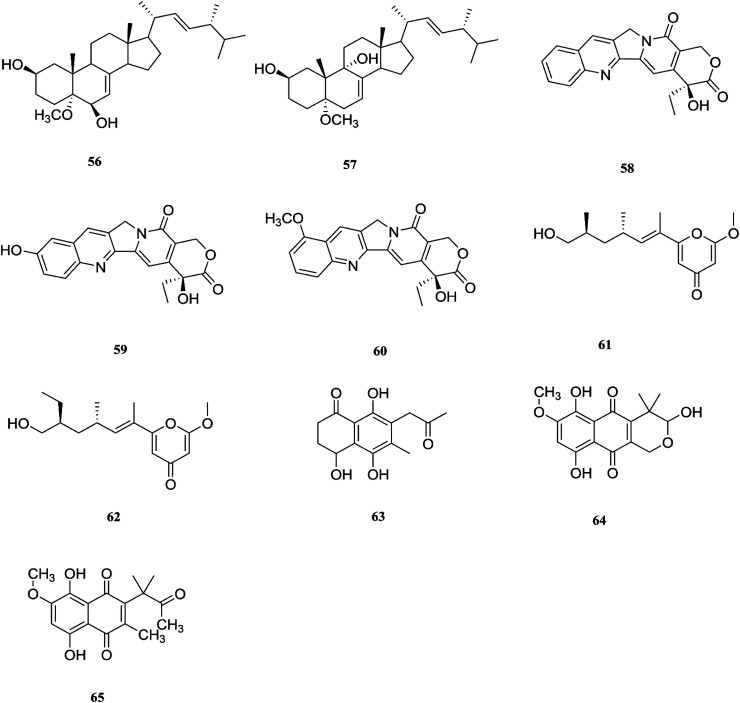
Various biologically active compounds (56–65) isolated from *F. solani*.

Camptothecin inhibits the intra-nuclear enzyme topoisomerase-I, required for DNA replication and transcription, but 10-hydroxycamptothecin has been shown to act against a broad spectrum of cancers.^34^ These results strongly indicate that *Fusarium* spp. isolated from medicinal plants may serve as a potential source of anticancer compounds with multiple modes of action. Further investigation to isolate these active compounds in good yields followed by mode of action studies could reveal new potential drugs against multiple cancer diseases. The water-soluble salt camptothecin–sodium is highly toxic in animals, whereas its semisynthetic derivatives irinotecan and topotecan do not cause haemorrhagic cystitis because of their higher physicochemical stability and solubility at lower pH values, although this may cause myelosuppression, neutropenia and, to a lesser extent, thrombocytopenia, which are dose-limiting toxic effects of topotecan.^35^ The chemical investigation of *F. solani* JS-0169 found in *Morus alba* leaves resulted in six compounds, including: one new γ-pyrone, 6-((9*R*,11*R*,*E*)-13-hydroxy-9,11-dimethyloct-7-en-7-yl)-2-methoxy-4*H*-pyran-4-one (61), a known γ-pyrone, fusarester D (62), and two known naphthoquinones, karuquinone B (63) and solaniol (49).^25^ 3,6,9-trihydroxy-7-methoxy-4,4-dimethyl-3,4-dihydro-1*H*-benzo[*g*]isochromene-5,10-dione (64), 3-*O*-methylfusarubin (65) and fusarubin (27) were isolated from *F. solani* present in *Glycyrrhiza glabra* (Liquorice). These compounds were found to exert antimicrobial activity against various bacterial strains, such as *E. coli*, *S. pyogenes*, *Klebsiella*, *B. cereus*, and *S. aureus* (MIC: <1 to 256 μg mL^−1^), besides their inhibition against *Mycobacterium tuberculosis* strain H37Rv (MIC: 8–256 μg mL^−1^).^36^ Further investigation of these compounds could lead to new antibiotics with multi-target activity; this feature is needed to prevent the future development of pathogen resistance to new drugs. The chemical structures are shown in Fig. 7 and 8.

### 2.3. F. proliferatum

**Fig. 9:**
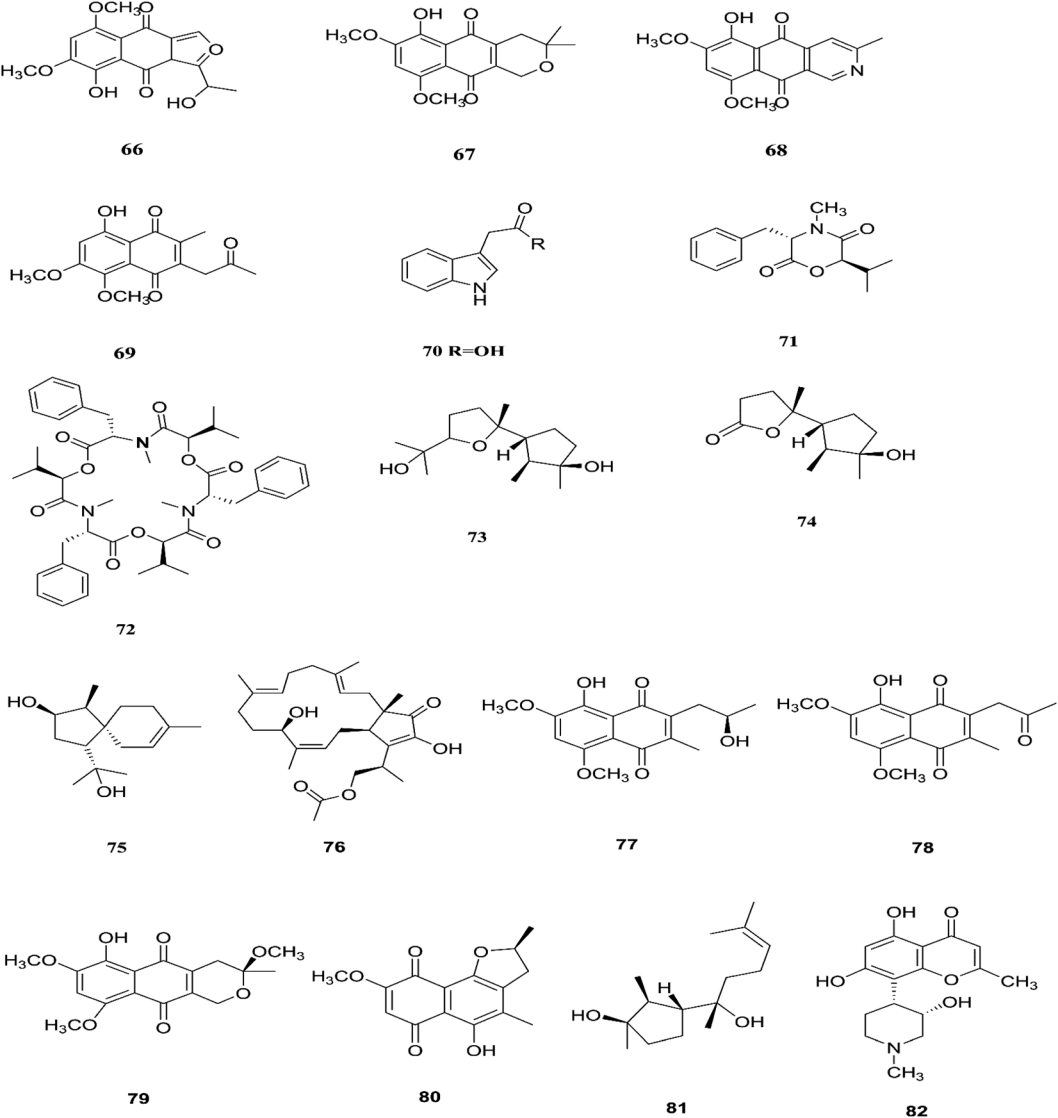
Various biologically active compounds (66–82) isolated from *F. proliferatum*.

*F. proliferatum*, which isolated from *Syzygium cordatum* was a source of several biologically active compounds, including: ergosta-5,7,22-trien-3β-ol (14), nectriafurone-8-methyl ether (66), 9-*O*-methyl fusarubin (67), bostrycoidin (29), bostrycoidin-9-methyl ether (68) and 8-hydroxy-5,6-dimethoxy-2-methyl-3-(2-oxo-propyl)-1,4-naphthoquinone (69), two of which were also identified in many *Fusarium* species such as *F. oxysporum* and *F. solani*.^37^ All these compounds exhibited 100% cytotoxic activity against *Artemia salina*, brine shrimp, at 100 μg mL^−1^.^37^ Three alkaloids identified as indol-3-acetic acid (70), methyl indolyl-3-acetate (2), and bassiatin (71), together with depsipeptide, known as beauvericin (72), three sesquiterpenoids identified as epicyclonerodiol oxide (73), cyclonerodiol lactone (74), and 3β-hydroxy-β-acorenol (75), a sesterterpene fusaproliferin (76), four 1,4-naphthoquinones, 5-*O*-methylsolaniol (77), 5-*O*-methyljavanicin (78), methyl ether fusarubin (79) and anhydrojavanicin (80), and (1*R*,2*S*,3*R*)-3-((*S*)-2-hydroxy-6-methylhept-5-en-2-yl)-1,2 dimethylcyclopentan-1-ol (81) were isolated from the derived fungus *F. proliferatum* AF-04 extract of the green Chinese onion.^38^ Compounds (73, 74, and 77–80) showed selective potent antibacterial activity toward various pathogenic bacteria including *B. megaterium*, *B. subtilis*, *C. perfringens*, *E. coli*, MRSA, and RN4220.^39^ Compound 79 displayed an MIC of 50.0 μg mL^−1^ against *B. megaterium*, which was similar to that ampicillin and erythromycin, and stronger than streptomycin.^39^ On the other hand, none of the tested compounds showed any significant inhibition of the strains Mu50 and Newman WTsrains.^39^ Rohitukine (82) is a chromone alkaloid that possesses anti-inflammatory, anti-cancer and immuno-modulatory properties isolated from the endophytic fungus *Fusarium proliferatum* (MTCC 9690) found in the inner bark tissue of *Dysoxylum binectariferum* Hook. f. (Meliaceae).^40^ The crude methanolic extract of *F. proliferatum* was found to be cytotoxic against HCT-116 and MCF-7 human cancer cell lines (IC_50_ = 10 μg mL^−1^), compared to camptothecin as a positive control with an IC_50_ of 1 μg mL^−1^.^40^ It also displayed promising activity in the HL-60 and Molt-4 (leukemia) cell lines with GI_50_ values of 10 and 12 μM, respectively. It showed inhibition of Cdk2/A and Cdk9/T1 with IC_50_ values of 7.3 and 0.3 μM, respectively. It was observed that rohitukine binds to the ATP binding site of the Cdk9/cyclin T1 complex by a dense network of H-bonding and van der Waals interactions that overall does not allow binding of substrate ATP to Cdk_9_/cyclin T_1_. The carbonyl group of rohitukine interacts with the NH group of Cys 106 and the piperidinyl 3-hydroxyl group interacts with the Lys-48 side chain of the hinge region with a plethora of hydrogen bonds. The piperidinyl *N*-methyl group of rohitukine easily gets protonated due to ionization, therefore the protonated piperidinyl NH^+^ interacts with the side chain Asp 167 of the DFG signature.^41^ Rohitukine possesses only a small methyl group, which leads to a loss of hydrophobic interactions and subsequently its Cdk9 inhibition potency.^41^ The chemical structures of *F. proliferatum* are shown in Fig. 9.

### 2.4. F. napiforme

**Fig. 10:**
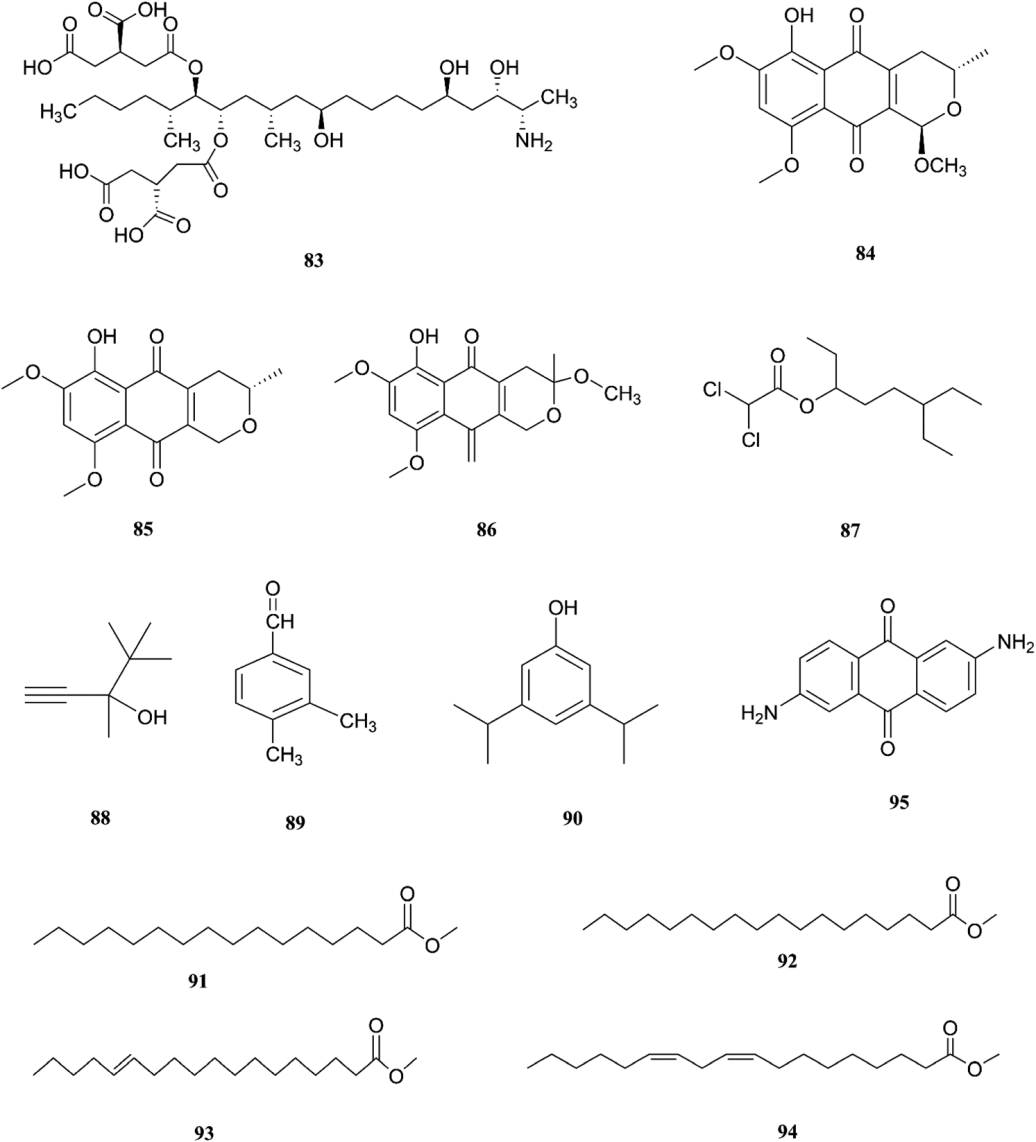
Various biologically active compounds (83–95) isolated from *F. napiforme*.

*F. napiforme* was first isolated from millet and sorghum from Southern Africa, and soil debris from grassland in Australia; it is a source of fumonisin B1 (83), and fumonisins are believed to cause toxicity by blocking ceramide synthase, a key enzyme in sphingolipid biochemistry that converts sphinganine (or sphingosine) and fatty acyl CoA to ceramide, which can cause fatal illnesses in some animals and is a suspected human esophageal carcinogen.^42,43^ On the other hand, the fungal culture of the IP-28 strain from the *Rhizophora mucronata* plant led to identification of two new naphthoquinone derivatives, 6-hydroxyastropaquinone B (84) and astropaquinone D (85), with a known compound, 3-*O*-methyl-9-*O*-methyl fusarubin (86).^44^ Compounds (84–86) exhibited moderate activity against *S. aureus* and *P. aeruginosa* with MIC values of 6.3, 12.5, and 6.3 μg mL^−1^ and 6.3, 6.3, and 6.3 μg mL^−1^, respectively, with no effect on *Aspergillus clavatus* nor *C. albicans* (at 25 μg mL^−1^) and phytotoxic action on lettuce seeding at a concentration of 30 μg mL^−1^.^44^ The GC/MS analysis of the crude extract of *Psidium guajava* leaves showed the presence of different compounds, including: dichloroacetic acid, 6-ethyl-3-octyl ester (87), 3,4,4-trimethyl-1-pentyn-3-ol (88), benzaldehyde-3,4-dimethyl (89), phenol-3,5-bis(1,1-dimethylethyl) (90), hexadecanoic acid-methyl ester (91), methyl stearate (92), *trans*-13-octadecenoic acid-methyl ester (93), 9,12-octadecadienoic acid-methyl ester (94), and 2,6-diaminoanthraquinone (95).^45^ The crude extract showed 69.74 ± 0.49% mean cytotoxicity against the A549 cell line; it also exhibited potent antioxidant potential with IC_50_ values of 299.4 μg mL^−1^ against the DPPH radical, 492.8 μg mL^−1^ against the phosphomolybdate ion, and 204.6 μg mL^−1^ against Ferric reducing power. These results strongly proved its ability to be a potential source for the formulation of natural anticancer and antioxidant drugs.^45^ The chemical structures of *F. napiforme* are shown in Fig. 10.

### 2.5. F. lateritium

**Fig. 11:**
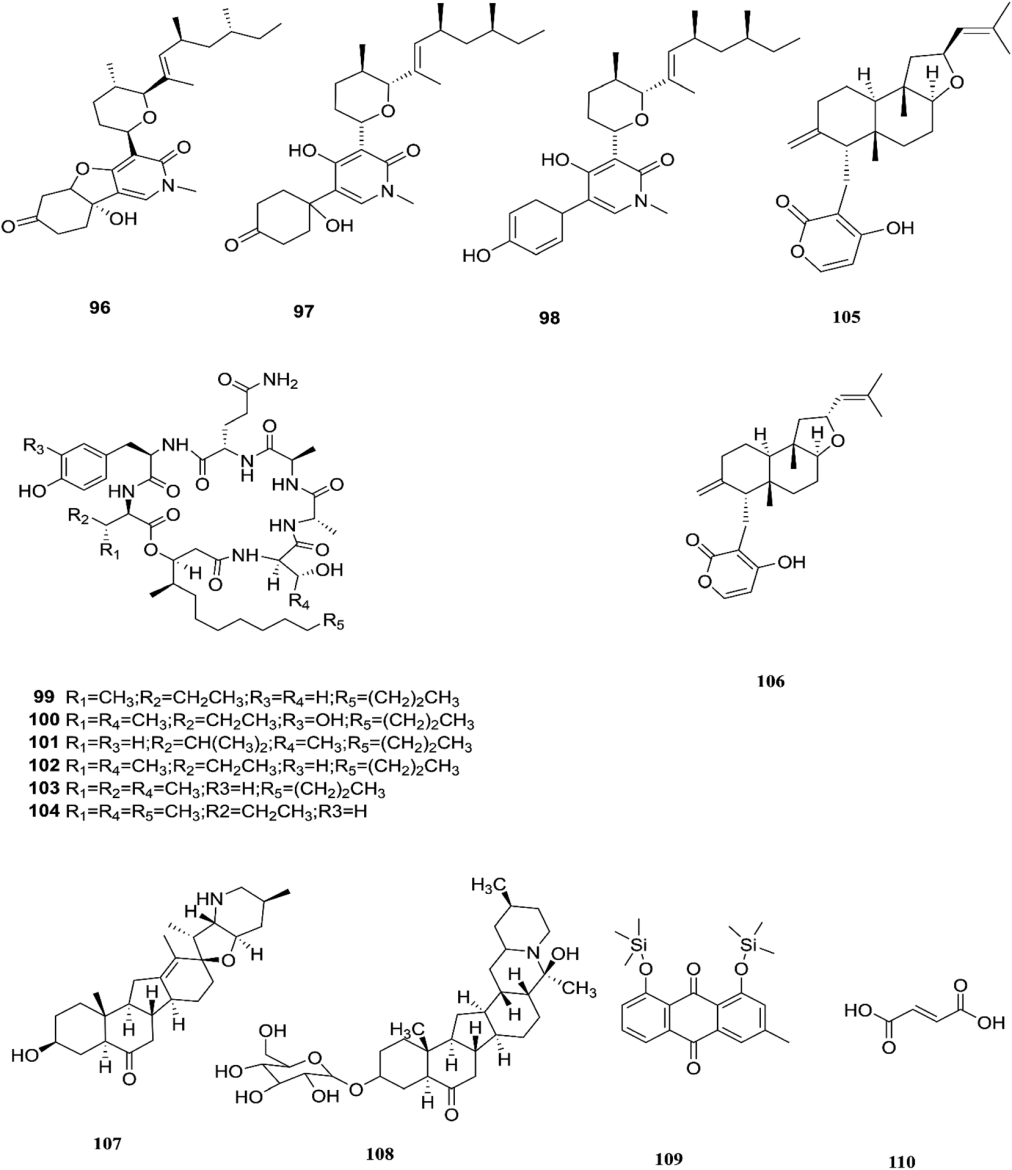
Various biologically active compounds (96–110) isolated from *F. lateritium*, *F. subglutinans* and *F. redolens*.

Three tricyclic pyridone alkaloids, identified as 6-deoxyoysporidinone (96), 4,6′ anhydrooxysporidinone (97) and sambutoxin (98), were separated from *F. lateritium* associated with Cornus officinalis fruits.^46^ 4,6′-Anhydrooxysporidinone was shown to protect HT22 cells through several mechanisms such as the inhibition of glutamate-induced cytotoxicity, accumulation of intracellular reactive oxygen species, increasing superoxide anion (Ca^2+^) production, and depolarization of the potential of the mitochondrial membrane.^46^ Additionally, its ability to inhibit cytochrome c release and cleave caspase-9 and -3 in glutamate-treated HT22 cells led to inhibition of apoptotic cell death.^46^ Two new cyclic lipopeptides, acuminatums E (99) and F (100), with four known cyclic lipopeptides, acuminatums A–D (101–104), were obtained from *Adenanthera pavonina* leaves, and these compounds exhibited antifungal activity against *Penicillium digitatum* with inhibition zones ranging from 1.5 mm to 9.0 mm in which *acuminatum* F (100) was the strongest with an inhibitory zone range of 6.5–9.0 mm.^47^ These investigations showed that *Fusarium* spp. can also produce compounds that can serve as a good starting point for the discovery of new antifungal drugs. Therefore, further investigations are needed for the discovery of new antifungal drugs. The number of compounds isolated from this species is very limited and they are shown with those of *F. subglutinans* in Fig. 11.

### 2.6. F. subglutinans

*F. subglutinans* harbored in *Tripterygium wilfordii* vine is a good source of natural promising new immunosuppressive drugs that can influence the immune system of animals, such as subglutinols A (105) and B (106), which are diterpene pyrones isolated from *Fusarium subglutinans* that are of interest in the identification of compounds useful in the treatment of patients undergoing organ transplantation to avoid rejection.^48^ Subglutinol A was effective in abolishing inflammatory cytokine production by skewed Th1 or Th17 CD4^+^ T cells, which is a hallmark of autoimmune disease.^49^ Subglutinols A and B are equipotent in the mixed lymphocyte reaction (MLR) assay and thymocyte proliferation (TP) assay (IC_50_ = 0.1 μm).^50^ Subglutinol A is an immunosuppressive drug without undesirable side effects to bone structure.^50^

### 2.7. F. redolens

The fungal strain 6WBY3 isolated from *Fritillaria unibracteata* var. *wabuensis* (fresh bulbus) yields two steroidal alkaloids, peimisine (107) and imperialine-3β-d-glucoside (108).^51^ Peimisine helps in getting rid of sputum and cough symptoms, it is considered to be a good lead for new anti-tumor drugs, and it exhibits potent inhibitory effects on angiotensin converting enzyme, due to its improvement in caspase-3 expression level, together with the significant inhibition of tumor angiogenesis and apoptosis induction.^51^*F. redolens* fermentation broth was able to produce taxol (24) from Himalayan Yew bark, whose presence in the fungal broth was determined by reverse-phase HPLC and mass spectroscopy, and it is considered to be a new source for the production of this important anticancer drug.^52^ The crude extract of *F. redolens* isolated from Olive (*Olea europaea* L.) stem resulted in the isolation of chrysophanol (109) and fumaric acid (110); chrysophanol is an anthraquinone having several pharmacological activities such as anticancer, hepatoprotective, neuroprotective, anti-inflammatory, and antiulcer, in addition to its antimicrobial potency against various microbial strains, including: *C. albicans*, *C. neoformans*, *T. mentagrophytes*, and *A. fumigatus*, with MIC values of 50, 50, 25, and 50 μg mL^−1^.^53^ Fumaric acid (110) is one of the more commonly produced secondary metabolites and it is used in the treatment of psoriasis or multiple sclerosis. Clinical studies in psoriasis showed a reduction of peripheral CD4^+^ and CD8^+^ T-lymphocytes due to the ability of fumaric acid esters to induce apoptosis.^53^ Fumaric acid esters may cause both renal Fanconi syndrome and acute kidney injury. This effect may be ameliorated by antagonism of the organic anion transporter with probenecid.^54^ Beauvericin (BEA) (72) was also obtained from this fungal endophyte isolated from rhizomes of the Chinese medicinal plant *Dioscorea zingiberensis*.^55^ Beauvericin causes cytotoxicity in several cell lines and has the ability to produce oxidative stress at the molecular level; its mechanism of action seems to be related to its ionophoric activity, which increases ion permeability in biological membranes.^56^ Moreover, BEA is genotoxic (causing DNA fragmentation, chromosomal aberrations and micronucleus) and causes apoptosis with the involvement of the mitochondrial pathway.^56^ Despite its strong cytotoxicity, no risk assessment has been carried out by authorities due to a lack of toxicity data.^56^ Beauvericin has the potential to be an antibacterial drug, with IC_50_ values between 18.4 and 70.7 μg mL^−1^ against six tested bacterial strains (*B. subtilis*, *S. haemolyticus*, *P. lachrymans*, *A. tumefaciens*, *E. coli* and *X. vesicatoria*).^55^ The chemical structures of *F. redolens* are shown in Fig. 11.

### 2.8. F. incarnatum

**Fig. 12:**
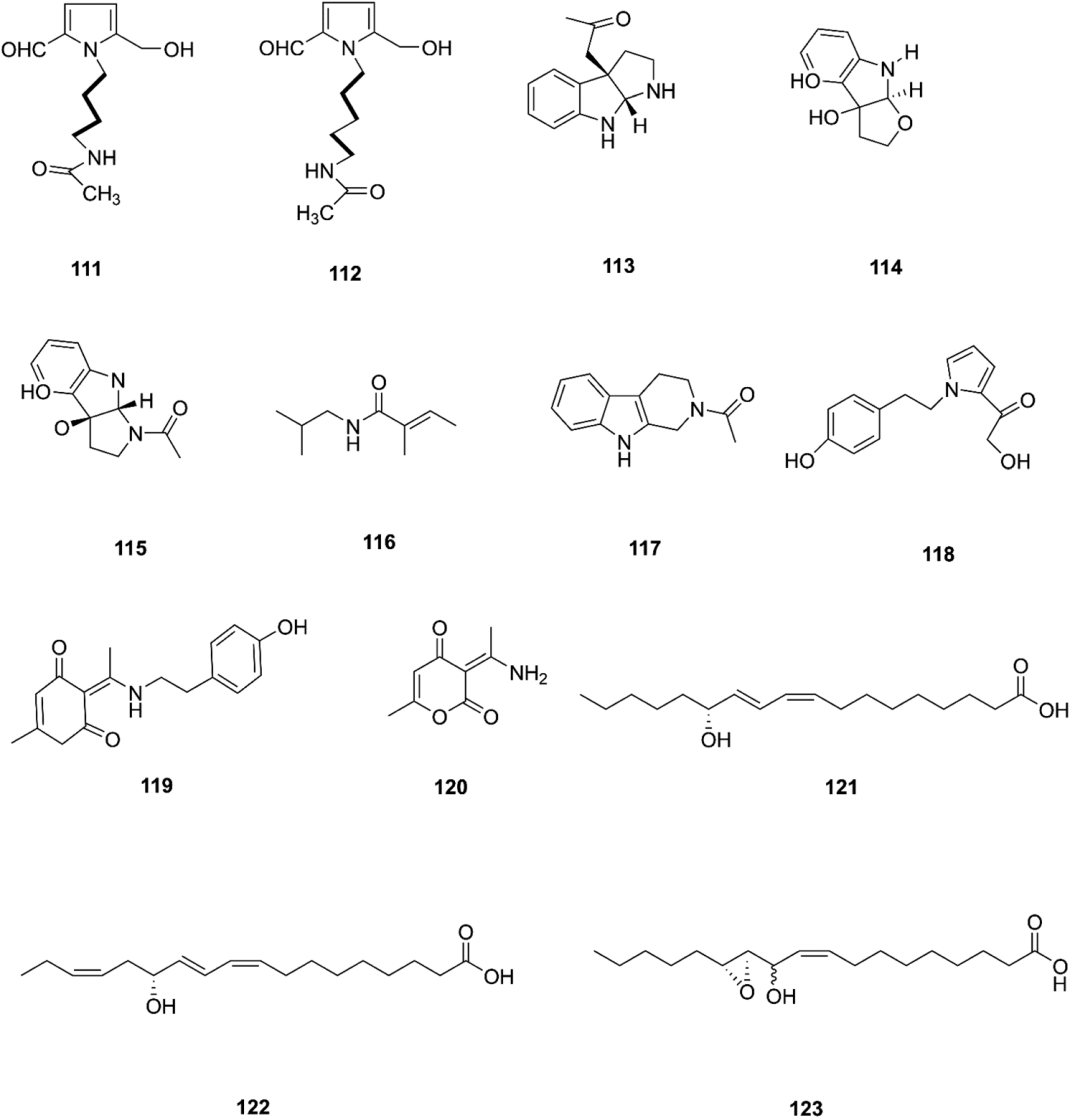
Various biologically active compounds (111–123) isolated from *F. incarnatum*.

Two new pyrrole alkaloids, *N*-[4-(2-formyl-5-hydroxymethyl-pyrrol-1-yl)-butyl]-acetamide (111) and *N*-[5-(2-formyl-5-hydroxymethyl-pyrrol-1-yl)-pentyl]-acetamide (112), together with a new indole derivative, (3*aR*,8*aR*)-3α-acetoxyl-1,2,3,3*a*,8,8*a*-hexahydropyrrolo-[2,3-*b*]indol (113), (−)-3α-hydroxyfuroindoline (114), (3*aR*,8*aS*)-1-acetyl-1,3,3*a*,8,8*a*-hexahydropyrrolo-[2,3-*b*]indol-3α-ol (115) and *N*-acetyltryptamine A (3), were isolated from *F. incarnatum* harbored in *Aegiceras corniculatum* leaves.^57^ Notably, these alkaloids exhibited weak activity against the cancer cell lines HeLa, K-562, and L-929 without showing any activity against *B. subtilis*, *S. aureus*, *E. coli*, and *C. albicans* (IC_50_ = 10 mg mL^−1^).^57^ Several unusual alkaloids were separated from the culture broth of *F. incarnatum* of the mangrove plant *Aegiceras corniculatum*, *N*-2-methylpropyl-2-methylbutenamide (116), 2-acetyl-1,2,3,4-tetrahydro-β-carboline (117), fusarine (118), fusamine (119), and 3-(1-aminoethylidene)-6-methyl-2*H*-pyran-2,4(3*H*)-dione (120).^58^ Only compounds 117, 119, and 120 showed weak antiproliferative and cytotoxic activity against HUVEC, K-562, and HeLa human cell lines with IC_50_ values of (41.1, 33.3, 23.8 μg mL^−1^), (37.3 37.6 23.3 μg mL^−1^), (68.2 9.0103.6 μg mL^−1^) respectively, compared to Imatinib (18.5, 0.17, 65.8 μg mL^−1^).^58^ The crude extract of a pure *F. incarnatum* culture residing in the embryo of viviparous propagule was analyzed by HPLC-MS, resulting in the observation of various highly lipophilic compounds such as coriolic acid (121), didehydrocoriolic acid (122) and *cis*-12,13-epoxy-11-hydroxyoctadec-9-enoic acid (123).^59^ Coriolic acid is a natural compound that inhibits the formation of mammospheres and induces BCSC apoptosis; it also decreases the subpopulation of CD^high^_44_/CD^low^_24_ cells, a cancer stem cell (CSC) phenotype, and specific genes related to CSCs, such as Nanog, Oct_4_, and CD_44_.^60^ Coriolic acid could be a novel compound to target breast cancer stem cells *via* regulation of c-Myc.^60^ The chemical structures of *F. incarnatum* are shown in Fig. 12.

### 2.9. F. sambucinum

Bioactivity-based phytochemical investigation of *F. sambucinum* TE-6L harbored in *Nicotiana tabacum* L. led to the discovery and separation of two new angularly prenylated indole alkaloids with pyrano[2,3-*g*] indole moieties, amoenamide C (124) and sclerotiamide B (125), and four known biosynthetic congeners, sclerotiamide (126), notoamide B (127), speramide A (128), and notoamide D (129).^61^ All these compounds displayed selective antibacterial activity with MIC values ranging from 1.0 to 32 μg mL^−1^.^61^ Amoenamide C (124) demonstrated potent activity against *P. aeruginosa* with a MIC value of 1 μg mL^−1^, which was better than that of the positive control chloromycetin (MIC = 4 μg mL^−1^).^61^ Furthermore, sclerotiamide showed stronger antibacterial activity (MIC values of 4, 4, 8, and 8 against *E. coli*, *M. luteus*, *P. aeruginosa*, and *R. solanacearum*, respectively).^61^ Likewise, compounds 125–127 displayed potent insecticidal activity with mortality rates of 70.2, 83.2, and 70.5% against *H. armigera*, respectively, using matrine as a positive control, which had an 87.4% mortality rate.^61^

### 2.10. F. sporotrichioides

**Fig. 13:**
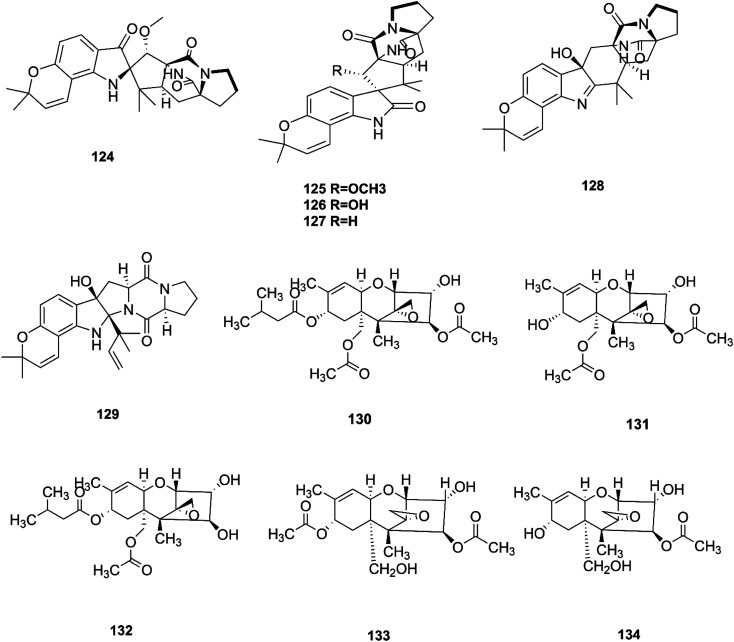
Various biologically active compounds (124–134) isolated from *F. sambucinum* and *F. sporotrichioides*.

T-2 toxin (130), neosolaniol (131) and HT-2 toxin (132) together with two new trichothecenes, 4β-8α-diacetoxy-12,13-epoxytrichothec-9-ene-3α,15-diol (133) and 4β-acetoxy-12,13-epoxy-trichothec-9-ene-3α,8α,15-triol (134), were isolated from the bean bull culture filtrate of the *F. sporotrichiides* M-1-1 strain.^62^ These compounds showed no antimicrobial activity, but this wasn't enough to classify them as inactive, therefore there is a need for further study of these compounds in comparison to others to reveal their outstanding potency.^62^ T-2 toxin is one of the most common toxic trichothecene mycotoxins due to its potent neurotoxicity.^63^ T-2 toxin can cross the blood–brain barrier due to its lipophilic nature and accumulate in the central nervous system (CNS) to cause neurotoxicity.^63^ The underlying mechanism is that T-2 toxin undergoes metabolism to produce epoxides that are extremely toxic compounds reacting with nucleophiles and promoting ROS production that mediate oxidative stress and mitochondrial dysfunction.^63^ These effects are compounded by the ability of T-2 toxin to deplete GSH in neuronal cells and brain tissues.^63^ Furthermore, exposure to T-2 toxin during pregnancy results in embryotoxicity and the abnormal development of offspring.^64^ The chemical structures of the isolated compounds of *F. sambucinum* and *F. sporotrichioides* are shown in Fig. 13.

### 2.11. F. tricinctum

*F. tricinctum* (Corda) Sacc. is a food contaminating mold that produces toxic metabolites with a wide distribution in crops and plant products.^65^ Two novel nor-sesquiterpenoids (135 and 136) containing a new tetrahydrofuran skeleton were separated from *F. tricinctum* harbored in the root of *Ligusticum chuanxiong*, and these compounds were elucidated as (*R*,2*E*,4*E*)-6-((2*S*,5*R*)-5-ethyltetrahydrofuran-2-yl)-6-hydroxy-4-methylhexa-2,4-dienoic acid (135) and (*S*,2*E*,4*E*)-6-((2*S*,5*R*)-5-ethyltetrahydrofuran-2-yl)-6-hydroxy4-methylhexa-2,4-dienoic acid (136). The cytotoxic potency of these compounds was evaluated by using MTT assay against HCT116, MCF-7, A549 and MV4-11 cancer cell lines, wherein compound (136) exhibited moderate growth inhibition against the MV4-11 cell line with an IC_50_ of 22.29 μM.^66^ The co-culture of *F. tricinctum* with *B. subtilis* 168 trpC2 on solid rice medium resulted in identification of the new compounds macrocarpon C (137), 2-(carboxymethylamino) benzoic acid (138), and (−)-citreoisocoumarinol (139) and (−)-citreoisocoumarin (140), and it also increased the accumulation of constitutively present secondary metabolites up to 78-fold including lateropyrone (141), cyclic depsipeptides of the enniatin type (142–144), and the lipopeptide fusaristatin A (145).^67^ Those compounds exhibited good antimicrobial activity against *B. subtilis*, *S. aureus*, *S. pneumoniae*, and *E. faecalis*, with MIC values ranging from 2 to 8 μg mL^−1^.^68^ On the other hand, the co-cultivation of *F. tricinctum* and *S. lividans* resulted in the accumulation of several new compounds, the dimeric naphthoquinones (146–149), fusatricinone A (146), fusatricinone B (147), fusatricinone C (148), fusatricinone D (149) and dihydrolateropyrone (150), together with the cryptic compounds zearalenone (151) and 7-hydroxy-2-(2-hydroxypropyl)-5-methylchromone (152) that were not detected in axenic fungal controls.^67^ Additionally, two new alkaloids, rigidiusculamide E (153) and [–(α-oxyisohexanoyl-*N*-methyl-leucyl)_2_–] (154), together with two known ones, (−)-oxysporidinone (155) and (−)-4,6′-anhydrooxysporidinone (97), were isolated from the EtOAc extract of the mycelia culture obtained from the healthy root of *P. notoginseng* (Araliaceae). Rigidiusculamide E (153) showed significant inhibitory activity toward nitric oxide (NO) production on the Murine macrophage cell line with an IC_50_ value of 18.10 ± 0.16 μM.^69^

Two new rare irregular sesquiterpenes, tricinonoic acid (156) and tricindiol (157), and two known furanopyrrolidones, NG-391 (47) and NG-393 (48), were isolated from the EtOAc extract of *F. tricinctum*, found in *Rumex hymenosepalus* root.^65^ Using the One Strain Many Compounds (OSMAC) approach on *F. tricinctum* resulted in an up to 80-fold increase in the accumulation of new natural compounds such as fusarielin J (158), fusarielin K (159) and fusarielin L (160) together with the known derivatives fusarielins A (161) and B (162).^70^ Although fusarielin J (158) exhibited cytotoxic activity against the human ovarian cancer cell line (A2780), with an IC_50_ value of 12.5 μM, the other fusarielins showed very weak activity (436 μM) in the same assay.^70^ Additionally, enniatins, which are a group of antibiotics with six-membered cyclic depsipeptides, formed by the union of three molecules of d-α-hydroxyisovaleric acid and three *N*-methyl-l-amino acids, were identified from the *F. tricinctum* Corda endophyte in the fruits of *Hordeum sativum* Jess and *Aristolochia paucinervis*, and they were named enniatins A (163), A1 (164), B (165), B1 (166), B2 (167) and Q (168).^71^ Among these, enniatin Q is a new analog of ENA and the occurrence of EN B2 was reported for the first time from this endophyte.^71^ Enniatins exhibited antimicrobial and cytotoxicity activities against human cells; they also had a limited hemolytic effect, yet were found to be toxic at low doses to nucleated human cells causing apoptosis, mitochondrial damage, and reactive oxygen species production. They also interact with bacterial lipids, causing low to no membrane permeabilization, but induced membrane depolarization and inhibition of macromolecule synthesis.^72^

**Fig. 14:**
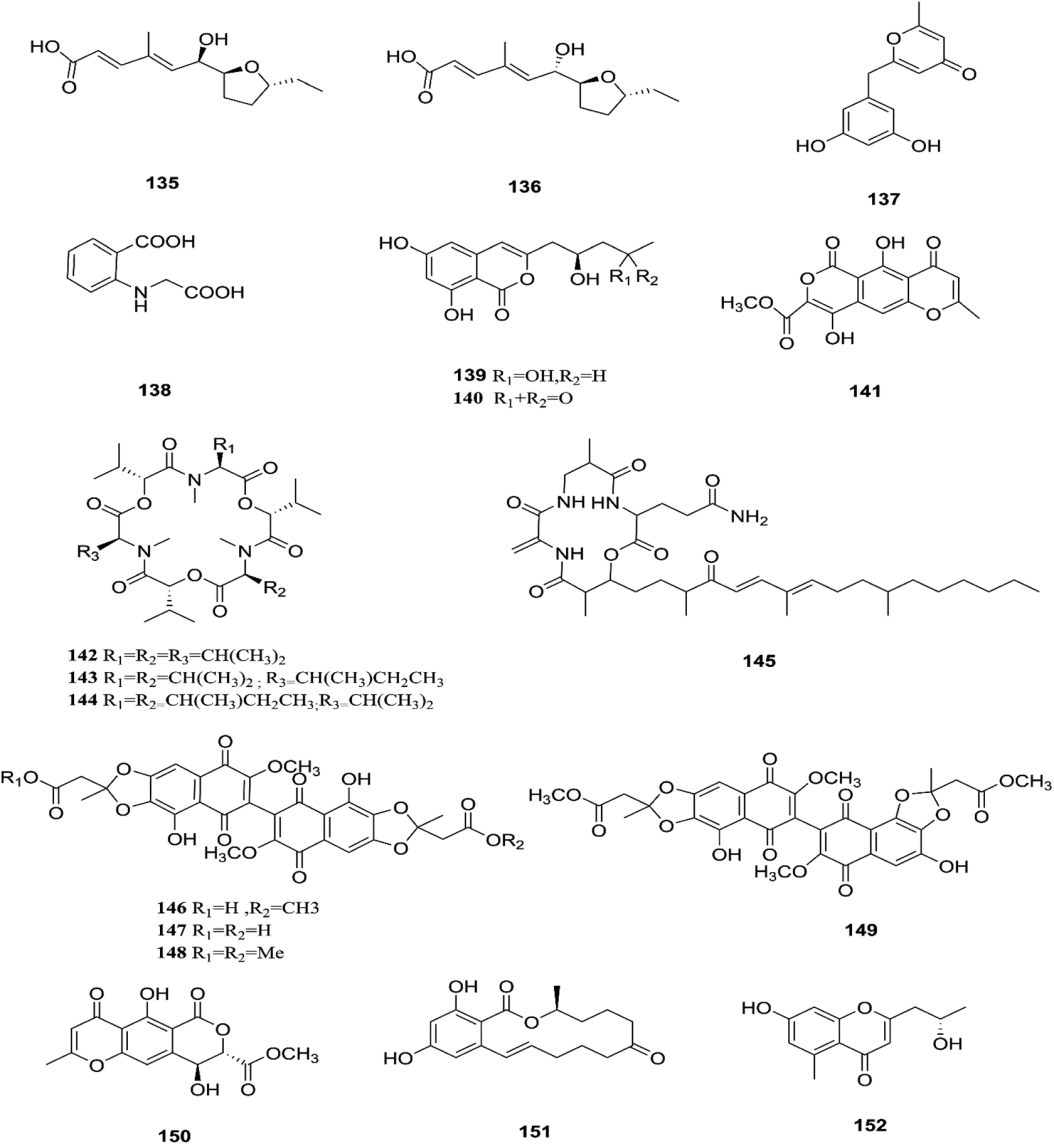
Various biologically active compounds (135–152) isolated from *F. tricinctum*.

**Fig. 15:**
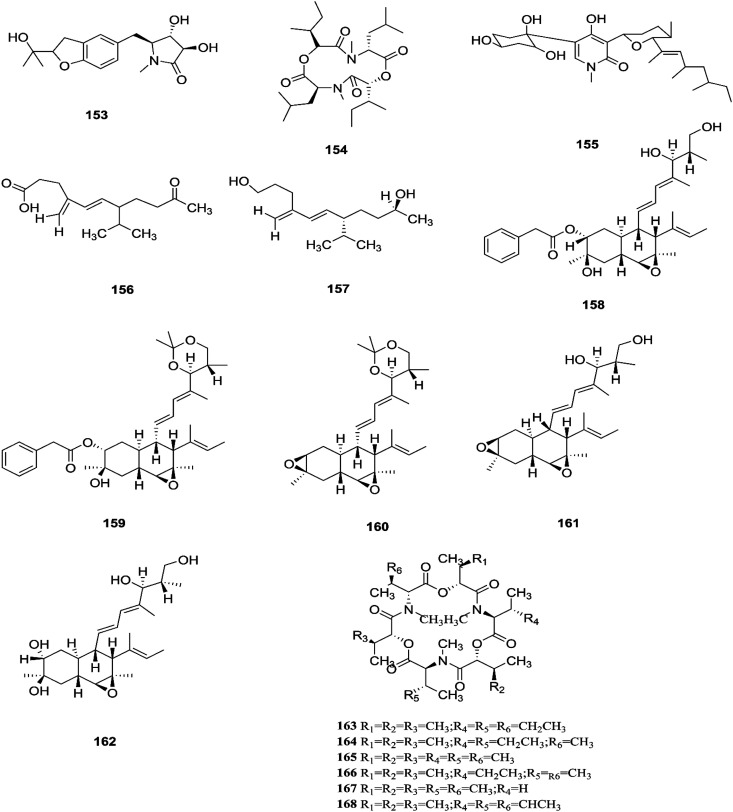
Various biologically active compounds (153–168) isolated from *F. tricinctum*.

**Fig. 16:**
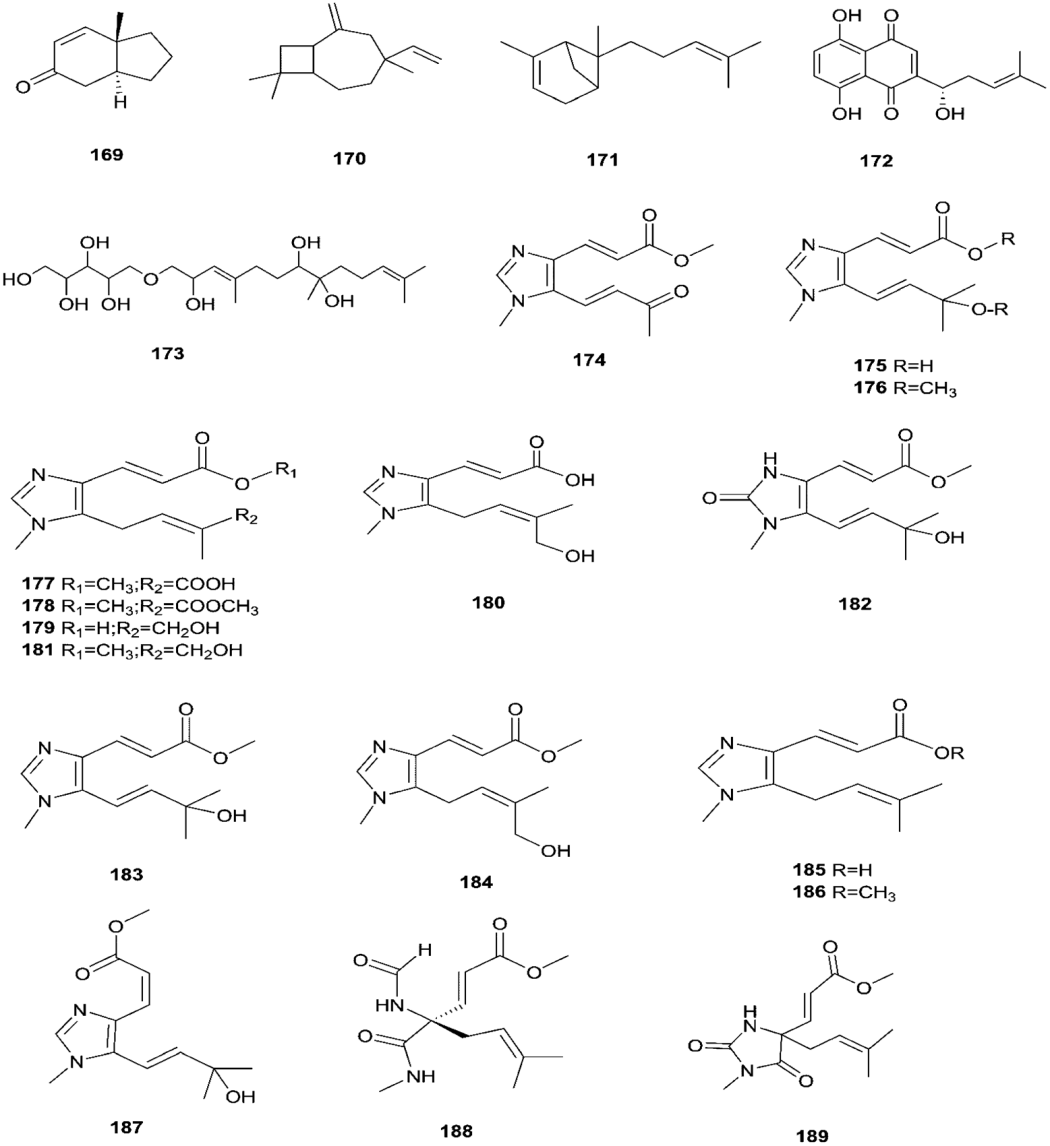
Various biologically active compounds (169–189) isolated from *F. tricinctum*.

Enniatins have hypolipidemic effects related to their ability to inhibit acyl coenzyme A cholesterol acyltransferase (ACAT) and triglyceride biosynthesis.^72^ The methanol extract of enniatins showed mild antileishmanial activity against *L. donovani* ATTC 39930D with the IC_50_ value of 16.96 μg mL^−1^ and the IC_90_ value of 30.4 μg mL^−1^.^71^ It also displayed moderate cytotoxic activity against HepG2 and C6 cells (IC_50_ values of 10–25 μM), and high toxicity against H4IIE cells (IC_50_ values of 1–2.5 μM).^71^ The hydro-distillation of *F. tricinctum* found in *Paris polyphylla* var *yunnanensis* resulted in a volatile oil, which when analyzed by gas chromatography-mass spectrometry (GC-MS) consisted of *trans*-1,2,3,3*a*,4,7*a*-hexahydro-7*a*-methyl-5*H*-inden-5-one (169), 2-methylene-4,8,8-trimethyl-4-vinyl bicyclo [5.2.0] nonane (170), and 2,6-dimethyl-6-(4-methyl-3-pentenyl) bicyclo [3.1.1] hept-2-ene (171).^73^ The volatile oil exhibited antimicrobial activity against eight bacterial strains, *A. tumefaciens*, *E. coli*, *P. lachrymans*, *S. typhimurium*, *X. vesicatoria*, *B. subtilis*, *S. aureus* and *S. haemolyticus*, with varied MIC values ranging from 25 to 45 μg mL^−1^ and 100 to 225 μg mL^−1^ against *C. albicans* and *M. oryzae*, respectively.^73^ Shikonin (172) is a naphthoquinone that was found to be produced only by *F. tricinctum* harbored in *Lithospermum officinale* L. roots. (family: Boraginaceae).^74^ Shikonin exhibited potent antibacterial activity against Gram-positive bacteria such as *B. subtilis*, *E. faecium*, and *S. aureus* at MIC values ranging from 0.30 to 6.25 mg mL^−1^.^74^ In contrast, it showed no activity against Gram-negative strains such as *M. luteus*, *E. coli*, and *P. aeruginosa*; shikonin exhibited potent anti-HCV activity, with an effective concentration (EC_50_) of 25 ng mL^−1^, which was far lower than that of the positive control ribavirin (EC_50_ = 2.6 μg mL^−1^).^74^ Another study on shikonin and its derivatives revealed its strong cytotoxic potency in various cancer cell lines (cervical cancer cell line HeLa, colon cancer cell line Hct116, hepatocellular carcinoma cell line Hep3B, and a lung cancer cell line (A549)) having a 50% growth inhibition range of 0.5–3.0 μM.^74^ Shikonin is known to suppress proliferation and induce apoptosis in a variety of cancer cell lines by inhibiting cell cycle progression, disrupting Ca^2+^ homeostasis, inducing oxidative stress and triggering mitochondrial dysfunction. It also activates caspases-3, -8 and -9, and K^+^ efflux, and regulates Bax, Bcl-2, p_53_ and caspase-3 expression.^75^ Likewise, new sesquiterpenoid ethers with unique skeletons obtained from *F. tricinctum* of *Salicornia bigelovii* were named fusartricin (173) and fusarielin B (162) and displayed noticeable antimicrobial potency against *E. aerogenes*, *M. tetragenu* and *C. albicans* with MIC values of 19, 19 and 19 μM, respectively.^76^ Furthermore, the study of one kiwi endophytic fungus, *F. tricinctum*, led to the separation of nine new imidazole alkaloids, fusaritricines A-I (174–182), together with seven known analogues (183–189), among which compounds 175, 176 and 182 and 186 showed good antibacterial activity against *P. syringae* pv. *actinidiae* (MIC values between 25–50 μg mL^−1^).^77^ It is clear from these investigations that *Fusarium* species are the source of not only broad-spectrum antimicrobial compounds but also compounds with specific activity against a target pathogen. The chemical structures of *F. tricinctum* are shown in Fig. 14–16.

### 2.12. F. equiseti

**Fig. 17:**
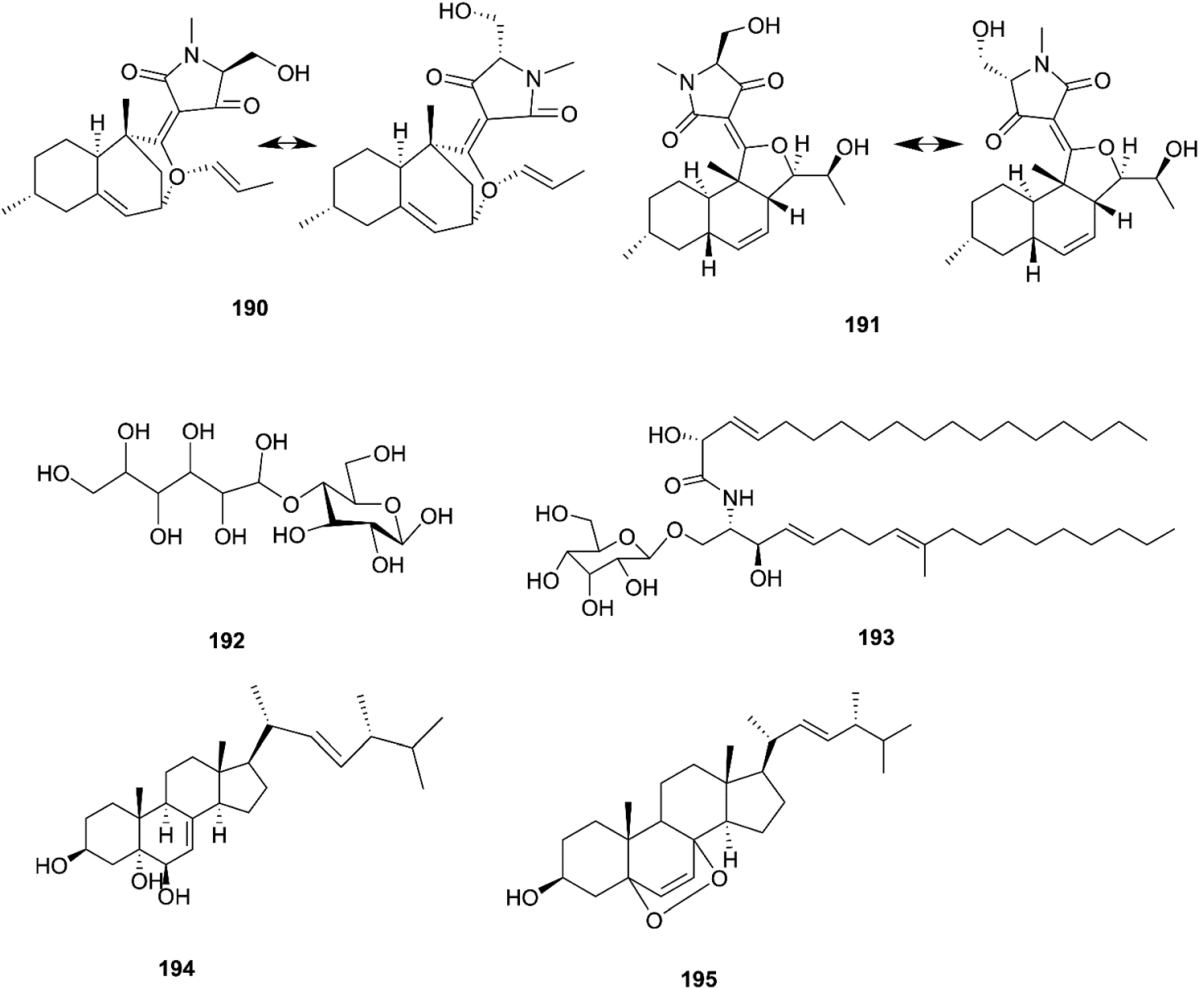
Various biologically active compounds (190–195) isolated from *F. equiseti*.

*F. equiseti* is a good source of various classes of secondary metabolites, as well as a pair of undescribed 3-decalinoyltetramic acid (3DTA) *E*/*Z* diastereomers, decalintetracids A (190) and B (191).^78^ Both of them are considered to be potential natural herbicides, owing to their phytotoxic potency toward *A. retroflexus* L. and *A. hybrid*.^78^ Additionally, a new glucitol, diglucotol (192), together with five known compounds, cerebroside C (193), Nb-acetyltryptamine (3), 3β,5α,9α-trihydroxy-(22*E*,24*R*)-ergosta-7,22-dien-6-one (31), cerevisterol (194) and ergosterol peroxide (195), was separated from *F. equiseti* found in *Salicornia bigelovii* Torr.^79^ Diglucotol (192) displayed weak cytotoxic potency toward MCF-7, MDA-MB-231 and Caco-2 cancer cells with EC_50_ values of 97.56, 92.35 and 99.39 μM, respectively.^79^ Whereas, cerevisterol (CRVS) (194) showed higher potency against the same cancer cell lines with EC_50_ values of 32.4, 41.5 and 37.56 μM, respectively.^79^ Consequently, ergosterol peroxide was less potent than cerevisterol with EC_50_ values of 64.5, 52.4 and 77.56 μM, respectively, while all the other compounds exhibited no activity.^79^ Cerevisterol is a natural agent for treating inflammatory disease. It suppresses the LPS-induced production of NO and PGE_2_, likely by reducing the expression of iNOS and COX-2. CRVS also decreases the expression of pro-inflammatory cytokines, such as TNF-α, IL-6, and IL-1β. CRVS halted the nuclear translocation of NF-κB by blocking the phosphorylation of inhibitory protein κBα (IκBα) and suppressing NF-κB transactivation. It also suppresses the mitogen-activated protein kinase (MAPK) signaling pathways. CRVS treatment also inhibited the transactivation of AP-1 and the phosphorylation of c-Fos.^80^ Their chemical structures are shown in Fig. 17.

### 2.13. F. oxysporum

**Fig. 18:**
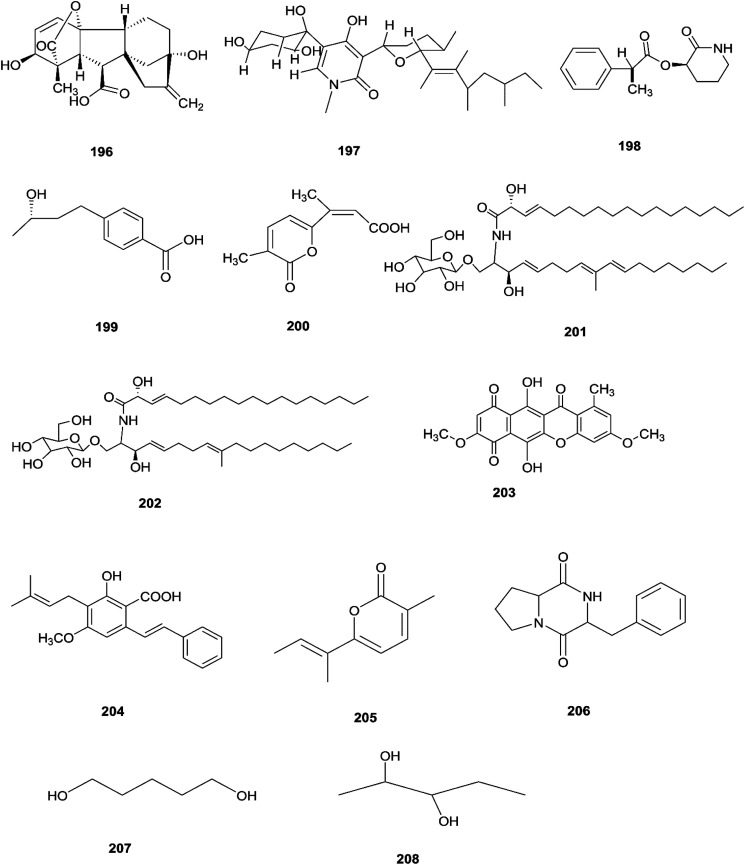
Various biologically active compounds (196–208) isolated from *F. Oxysporum*.

The *F. oxysporum* strain, found in *Coriandrum sativum* root, was able to exhibit maximal production of gibberellic acid (196), which is a diterpenoid of the gibberellin family and its importance comes from the regulation of plant growth, and stimulation of numerous development processes.^81^ A new oxysporidinone analogue (4′-hydroxyl derivative of oxysporidinone) (197) and a new 3-hydroxyl-2-piperidinone derivative 2-phenylpropionyl-2-piperidinone-3(*R*)-yl ester (198), along with the known compounds (−)-4,6′-anhydrooxysporidinone (97), (+)-fusarinolic acid (199), gibepyrone D (200), beauvercin (72), cerevisterol (194), fusaruside (201), and (2*S*,2′*R*,3*R*,3′*E*,4*E*,8*E*)-1-*O*-d-glucopyranosyl-2-*N*-(2′hydroxy-3′-octadecenoyl)-3-hydroxy-9-methyl-4,8 sphingadienine (202) were separated from *F. oxysporum* harbored in the bark of *Cinnamomum kanehirae*.^82^ These compounds exhibited different cytotoxic potencies when using the MTT assay against various cancer cell lines such as PC-3, PANC-1, and A549.^82^ Furthermore, beauvericin (72) that was also isolated from *F. redolens* showed IC_50_ values of 49.5 ± 3.8, 47.2 ± 2.9, and 10.4 ± 1.6 μM, respectively, and also displayed anti-bacterial activity toward methicillin resistant *S. aureus* (MIC = 3.125 μg mL^−1^) and *B. subtilis* (MIC = 3.125 μg mL^−1^).^82^ Bikaverin (203) isolated from *F. oxysporum*, an endophyte of *Cylindropuntia echinocarpus*, showed cytotoxic activity against four cancer cell lines, NCI-H460, MIA Pa Ca-2, MCF-7, and SF-268. It also suppressed metastatic (PC-3M) and MDA-MB-231 cells and also displayed anti-angiogenic activity in HUVEC-2 cells at sub-lethal concentrations.^62^ Additionally, there is a natural antioxidant compound named cajaninstilbene acid (204) that was isolated from different *Fusarium* species such as *F. oxysporum* (ERP-10), *F. solani* (ERP-07) and *F. proliferatum* endophytes of *Cajanus cajan*.^62^ The *F. oxysporum* extract showed strong anti-mutagenic activity and was able to produce gibepyrone A (205), pyrrolo [1,2-*a*] pyrazine-1,4-dione, hexahydro-3-(2-methylpropyl) (206) and indole-acetic acid (70) as major components.^62^ Notably, 1,5-pentanediol (207) and 2,3-pentanediol (208) reportedly obtained from *F. oxysporum* of *Curcuma amada* displayed anti-aging properties against *Caenorhabditis elegans*.^62^ Likewise, anhydrofusarubin (26) isolated from the *F. oxysporum* SS46 endophyte of *Smallanthus sonchifolius* showed promising potency against *L. braziliensis*.^83^ Their chemical structures are shown in Fig. 18.

### 2.14 *Fusarium* sp.

**Fig. 19:**
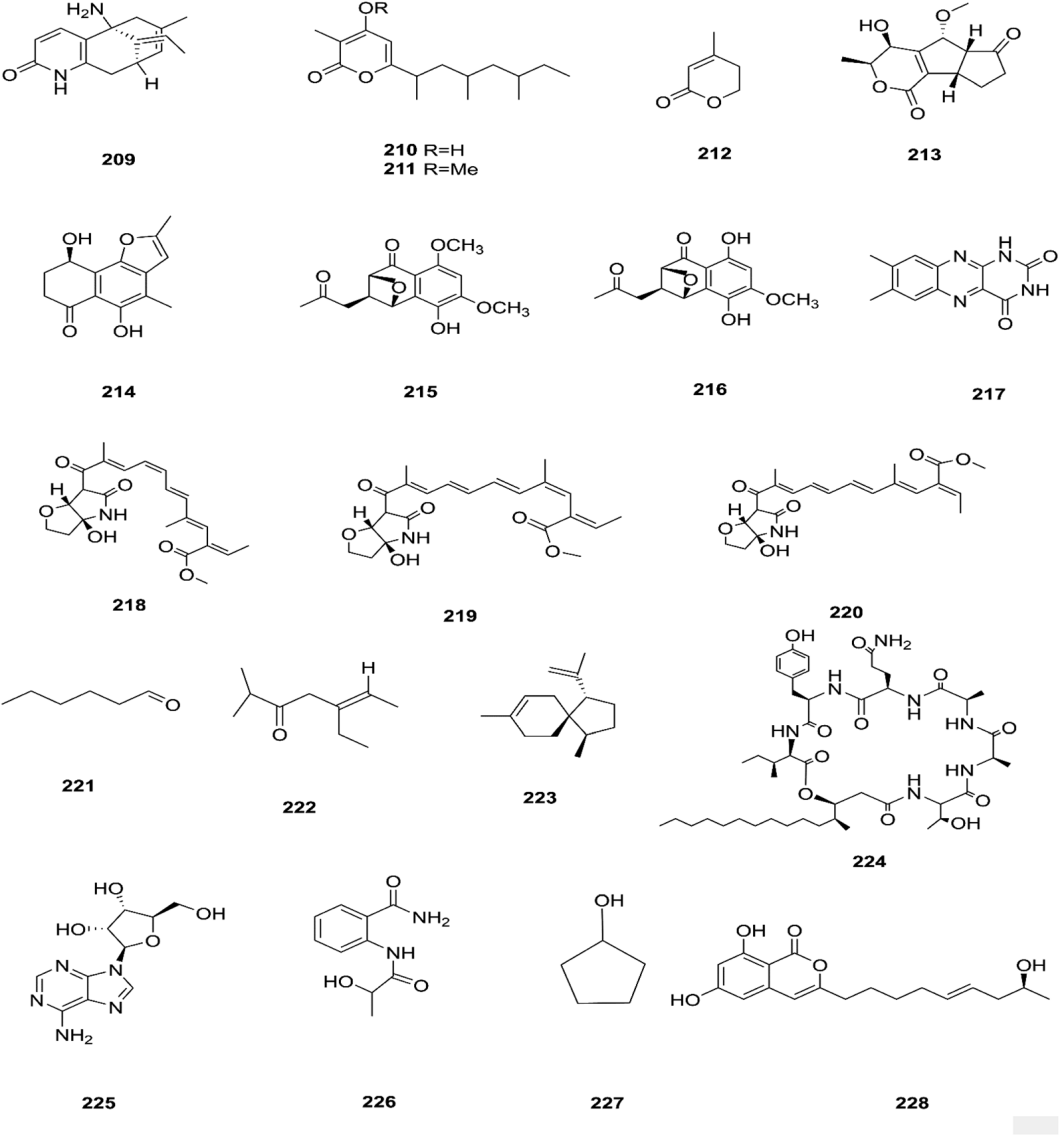
Various biologically active compounds (209–228) isolated from *Fusarium* sp.

There are several *Fusarium* species that remain unidentified although isolated and considered a source of many metabolites with exciting biological activities.^84^ Huperzine A (HupA) (209) was reportedly obtained from the *Fusarium* sp. Rsp5.2 strain cultivated from *Huperzia serrata*.^84^ HupA is a Lycopodium alkaloid, with high selectivity and potency as an acetylcholine esterase (AChE) inhibitor with an IC_50_ value of 2.849 ± 0.0026 μg mL^−1^. It is also used to control Alzheimer's disease owing to its unique pharmacological activities and low toxicity.^84^ This compound with strong activity and a low molecular weight is a good candidate for further optimization to develop new and more potent acetylcholinesterase inhibitors. HupA has potent anti-inflammatory activity by decreasing IL-1β and TNF-α protein expression, and suppressing transcriptional activation of NF-κB signaling. Thus, it provides protection from excitotoxicity and neuronal death as well as increasing GABAergic transmission associated with anticonvulsant activity.^85^ Two new α-pyrones, fupyrones A and B (210 and 211), and the known α-pyrone 4-methyl-5,6-dihydro-2*H*-pyran-2-one (212) were isolated from *Fusarium* sp. F20, harbored in the stems of *Mahonia fortune* (Chinese medicinal plant).^86^ These compounds showed no activity, neither antibacterial nor quorum sensing (QS) inhibitory against *C. violaceum*.^86^ A novel pyrone derivative, pysarone A (213), together with two new naphthalenone derivatives, 3-demethoxyl-fusarnaphthoquinone B (214) and (2*S*,3*S*,4*S*)-8-dehydroxy-8-methoxyl-dihydronaphthalenone (215), was obtained from *Fusarium* sp. HP-2, which was isolated from “Qi-Nan” agarwood, as well as two known compounds, (2*R*,3*S*,4*S*)-5,8-dihydroxy-6-methoxy-2-methyl-4-(l1-oxidanyl)-3-(2-oxopropyl)-3,4-dihydronaphthalen-1(2*H*)-one (216) and 7,8-dimethylbenzo[*g*]pteridine-2,4(1*H*,3*H*)-dione (217).^87^ However, only compound 215 showed weak AChE inhibitor activity with an 11.9% inhibition ratio at 50 μm mL^−1^.^87^ Interestingly, two new photosensitive geometrical isomers of lucilactaene 8(*Z*)-lucilactaene (218) and 4(*Z*)-lucilactaene (219) along with lucilactaene (220) and six other known compounds, fusarubin (27), (+)-solaniol (49), javanicin (54), 9-desmethylherbarine (51), NG-391 (47) and NG-393 (48), were obtained from *Fusarium* sp. QF001 isolated from *Scutellariae baicalensis* (inner rotten part of old roots).^88^ These compounds have the potential to be used as anti-inflammatory agents, because of their ability to inhibit NO production together with the expression of pro-inflammatory cytokines in LPS-stimulated macrophage cells.^88^ Exopolysaccharides (EPSs) are water-soluble high-molecular-weight carbohydrates with various pharmacological activities, such as antioxidant, immune-stimulatory, antitumor, hepato-protective, and anti-fatigue effects, as well as showing moderate *in vitro* antioxidant activity with moderate anti-proliferation in the HepG2 cell line.^89^ EPSs were isolated from the endophytic strain A14 derived from *Fritillaria unibracteata*, which is a herb most commonly used as an antitussive and expectorant drug in traditional Chinese medicine.^89^ Their chemical are structures shown in Fig. 19.

**Fig. 20:**
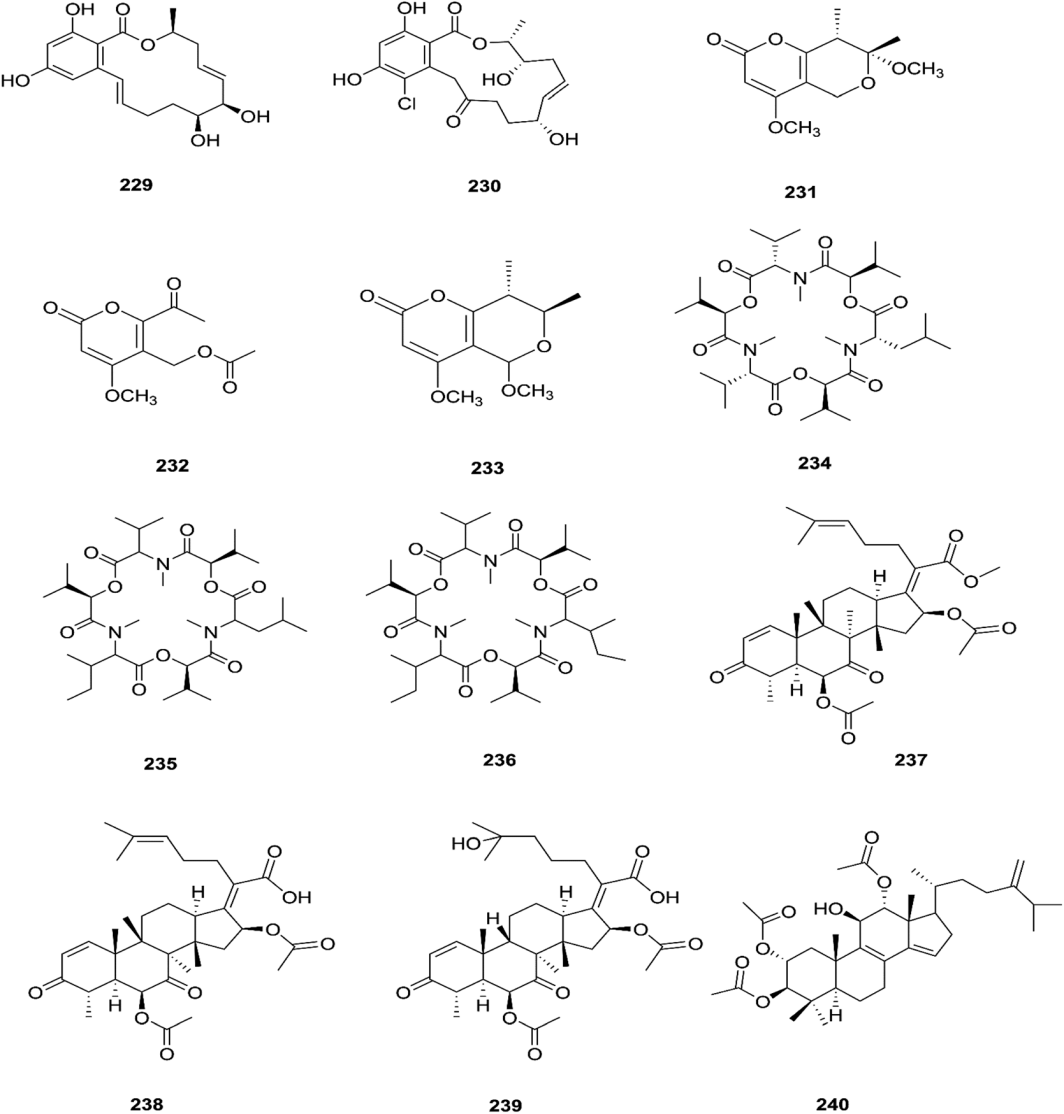
Various biologically active compounds (229–240) isolated from *Fusarium* sp.

The fungal endophyte *Fusarium* sp. isolated from *Monardacitriodora* Cerv. ex leaves showed a good ability to produce plant-like volatile organic compounds that are industrially important, in particular hexanal (221), (5*E*)-5-ethyl-2-methyl-5-hepten-3-one (222) and acoradiene (223) representing around 84.57% of the total VOCs.^90^ Additionally, *Fusarium* sp. isolated from *Mentha longifolia* L. (Labiatae) roots grown in Saudi Arabia produced cyclo depsipeptide fusaripeptide A (224), together with three known compounds, adenosine (225), 2[(2-hydroxypropionyl)amino]benzamide (226), and cyclopentanol (227).^91^ Notably, fusaripeptide A showed potent antifungal activity against *C. albicans*, *C. glabrata*, *C. krusei*, and *A. fumigates* with IC_50_ values of 0.11, 0.24, 0.19, and 0.14 μM, respectively. Fusaripeptide A serves as a good starting point for the discovery of new antifungal drugs. In addition, it showed significant anti-malarial activity toward *P. falciparum* (D6 clone) with an IC50 value of 0.34 μM.^91^ However, it showed potent cytotoxic activity toward L5178Y and moderate activity against PC_12_ with IC_50_ values of 5.71 and 9.55 μM, respectively, in comparison to doxorubicin (IC_50_ values of 4.60 and 5.71 μM, respectively).^91^ Likewise, *Fusarium* in (a new iso-coumarin derivative) (228) together with two known related resorcylic acid lactones, aigialomycin D (229) and pochonin N (230), was obtained from *Fusarium* sp. LN-10, originating from the leaves of *Melia azedarach*, and these compounds displayed significant toxicity toward brine shrimp larvae at a concentration of 10 μg mL^−1^ with mortality rates (%) of 78.2, 76.7 and 82.8, respectively, using chaetomugilin A as a positive control, which showed a mortality rate of 78.3% at the same concentration.^92^ The chemical investigation of the crude extract of *Fusarium* sp. 001 isolated from *Eupatorium adenophorum* resulted in the identification of two new chlamydosporol derivatives, fusarilactone A (231) and fusarilactone B (232), along with nine known compounds, *O*-methyl-isochlamydosporol (233), oxysporidinone (155), enniatin A (163), enniatin B (165), enniatin A1 (164), enniatin B1 (166), enniatin D (234), and a mixture of enniatin E1 (235) and enniatin E2 (236).^93^ Fusarilactone A showed mild cytotoxicity against three cancer cell lines (SMMC-7721, A-549, and MCF-7) with the IC_50_ varying between 17.5 and 35.1 μmol.^93^ Three helvolic acid derivatives named helvolic acid methyl ester, which is a new compound (237), helvolic acid (238), and hydrohelvolic acid (239) were isolated from *Fusarium* sp. in *Ficus carica* leaves and exhibited potent antifungal and antibacterial activities.^94^ Helvolic acid (238) showed potent cytotoxicity against different human cancer cells upon the co-administration of 10 mg per kg per day helvolic acid with 20 mg per kg per day cyclophosphamide (CTX)—a well-known chemotherapy drug—resulting in promising antitumor activity with a growth inhibitory rate of 70.90%, which was much higher than that of CTX alone (19.5%), and its mechanism of action may related to the Wnt/β-catenin signaling pathway.^95^ Their chemical structures are shown in Fig. 20.

Three new tetracyclic triterpenoids named integracide H (240), integracide I (241), and integracide J (242), along with integracide B (243) and integracide F (244), were obtained from *Fusarium* sp. isolated from *Mentha longifolia* L. (Labiatae) roots.^96^ Integracide H and integracide J showed significant activity against *L. donovani* with IC_50_ values of 4.75 and 3.29 μM, respectively, compared to pentamidine (IC_50_ 6.35 μM), and they also displayed potent cytotoxic activity toward BT-549, SKOV-3, and KB cell lines with IC_50_ values of 1.82, 1.32, and 0.18 μM, and 2.46, 3.01, and 2.54 μM, respectively.^96^ Fusaroside (245) is a unique trehalose-containing glycolipid composed of carboxylic carbon of a long-chain fatty acid attached to the 4-hydroxyl group of a trehalose unit isolated from the fermentation broths of *Fusarium* sp. LN-11 obtained from *Melia azedarach* leaves.^97^ Additionally, six known compounds, phalluside (246), (9R*,10R*,7*E*)-6,9,10-trihydroxyoctadec-7-enoic acid (247), porrigenic acid (248), (9*Z*)-2,3-dihydroxypropyl octadeca-9-enoate (249), cerevisterol (194), and ergokonin B (250), were isolated from this fungus.^97^ Fusarisetin E (251) and F (252), one chromone, fusarimone A (253), two benzofurans, fusarifurans A (254) and B (255), and three iso-coumarins, fusarimarins A–C (256–258), together with five analogues, equisetin (259), *epi*-equisetin (260), takanechromone B (261), altechromone A (262), 4*H*-1-benzopyran-4-one-2,3-dihydro-5-hydroxy-8-(hydroxylmethyl)-2-methyl (263), and aspergisocoumrin A (264), were isolated from *Fusarium* sp. 2ST2 of *Kandelia candel* leaves.^98^ Fusarisetin E and F showed significant cytotoxic activity against the A549 cell line, and compounds 263 and 264 showed potent activity against the A549 and MDA-MB-435 cell lines.^98^ A new isoflavone, 5-hydroxy-7-methoxy-4′-*O*-(3-methylbut-2-enyl) isoflavone (265), and five known compounds, eriodictyol (266), vittarin-B (267), 3,6,7-trihydroxy-1-methoxyxanthone (268), 1,3,6-trihydroxy-8-methylxanthone (269) and cyclo (Phe–Tyr) (270), were obtained from *Fusarium* sp. ZZF60 obtained from the leaves of the *Kandelia candel* tree.^99^ Whereas, two new cyclic depsipeptides, W493 C (271) and D (272), along with two known derivatives, W493 A (273) and B (274), were isolated from *Fusarium* sp. of *Ceriops tagal*.^100^ W493 A and B showed moderate antifungal activity against *Cladosporium cladosporiodes* with inhibition zones of 12.0 and 9.5 mm, respectively, using nystatin as a positive control, which exhibited a halo diameter of 26 mm. They also exhibited weak cytotoxic activity toward human ovarian carcinoma sensitive (A2780 sens) with growth inhibition values of 48% and 42%, respectively.^100^

**Fig. 21:**
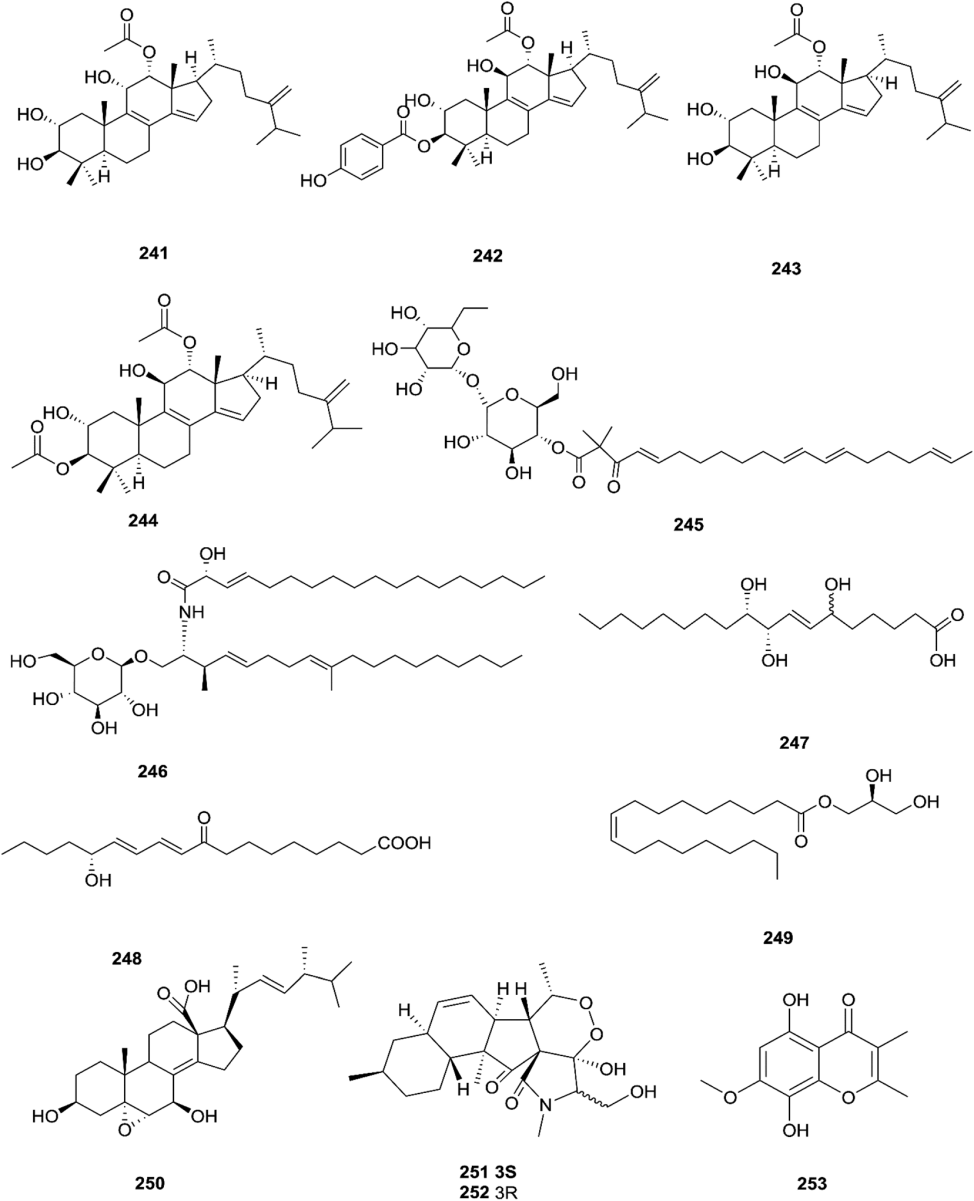
Various biologically active compounds (241–253) isolated from *Fusarium* sp.

**Fig. 22:**
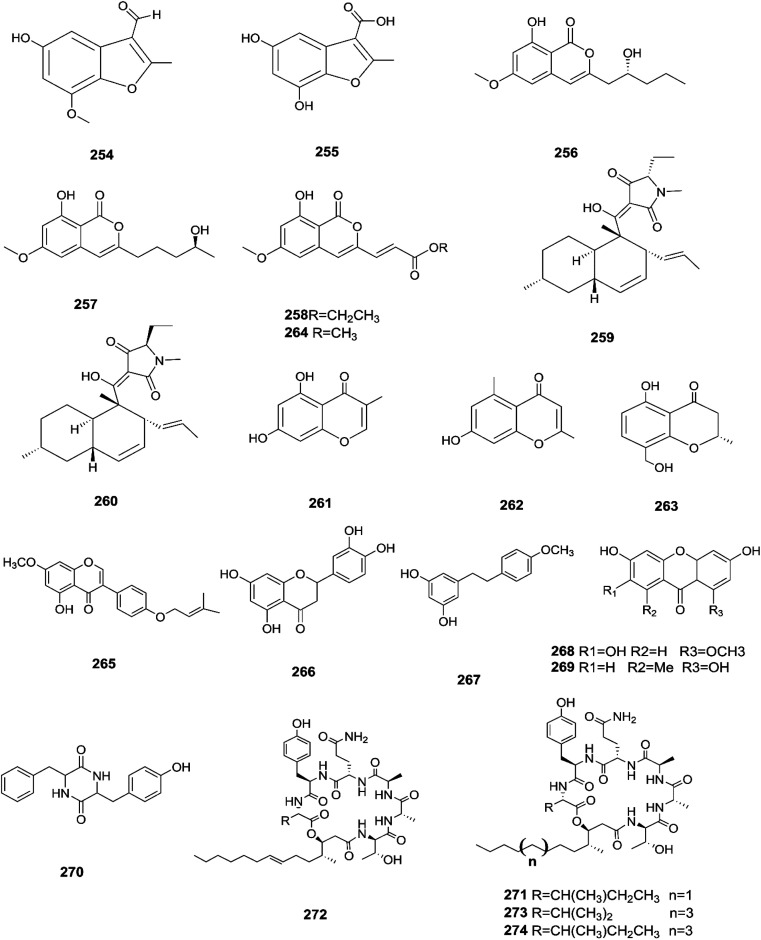
Various biologically active compounds (254–274) isolated from *Fusarium* sp.

The chemical structures of 241–274 are shown in Fig. 21 and 22. A new azaphilone derivative, named fusarone (275), was isolated from the fermentation broth of *Fusarium* sp. LN-12 obtained from *Melia azedarach* Linn. leaves.^101^ Likewise, a novel sulfur containing compound, fusaodavinvin (276), was isolated from *Fusarium* sp. (CTGU-ZL-34) found in *Davidia involucrate* leaves and stems together with 3-(2*R*-hydroxyl-1-one-propane)-indole (277), among which only fusaodavinvin exhibited significant cytotoxic activity against four cancer cell lines, A549, HepG2, Caski and MCF-7, with IC_50_ values of 11.5, 15.3, 15.2 and 60.5 μg mL^−1^, respectively.^102^ Consequently, two sterols, 5α,8α-epidioxyergosta-6,22-dien-3β-ol (278) and ergosta-8(9)22-dien-3β,5α,6β, 7-alpha-tetraol (279), and one fatty acid, butanedioic acid (280), were obtained from *Fusarium* sp. Ppf4 isolated from *Paris polyphylla* var. *yunnanensis* Hand.-Mazz rhizomes.^103^ Compound 278 was the most potent antimicrobial compound toward *X. veisicatoria* and *M. oryzae* with IC_50_ values of 86.7 and 92.8 μg mL^−1^, respectively.^103^ Ginsenoside (281) isolated from the *Fusarium* sp. PN8 endophyte of *Panax notoginseng* exhibited antibacterial activity (MIC 1.6–3.2 mg mL^−1^) against *E. coli*, *P. aeruginosa*, *B. subtilis*, *S. aureus* ATCC25923, and *S. lutea*, and two yeast strains (*C. albicans* and *S. cerevisiae*).^62^ It also showed anti-inflammatory action due to induction of the polarization of M1 and M2 macrophages and microglia and the contribution of M2-polarized cells to the suppression of inflammation progression and promotion of inflammation resolution.^104^ Fusarielin E (282), which is a new antifungal drug, was isolated from the culture broth of *F.* sp. CR377 separated from *Selaginella pallescens*, and it revealed significant biological activity against *Pyricularia oryzae* (MIC of 50 mg mL^−1^) compared to griseofulvin (MIC of 50 mg mL^−1^).^105^ Fusaruside (201) and (2*S*,2′*R*,3*R*,3′*E*,4*E*,8*E*)-1-*O*-β-d-glucopyranosyl-2-*N*-(2′-hydroxy-3′-octadecenoyl)-3-hydroxy-9-methyl-4,8-sphingadienine (202) isolated from *Fusarium* sp. IFB-121, an endophytic fungus in *Quercus variabilis*, showed strong antibacterial activity with MIC values of 1.9–7.8 μg mL^−1^.^62^ A new cyclic pentadepsipeptide, sansalvamide (283), separated from the surface of the seagrass *Halodule wrightii*, exhibited activity toward HCT-116 colon carcinoma with an IC_50_ value of 9.8 μg mL^−1^.^106^ Sansalvamide showed greater potency toward the colon cancer cell-line COLO 205 and melanoma cell-line SK-MEL-2 with IC_50_ values of 3.5 and 5.9 μg mL^−1^, respectively, compared to the antitumor agent mitomycin C, which exhibits an IC_50_ of 5.3 μg mL^−1^ toward these same two cell lines.^106^

**Fig. 23:**
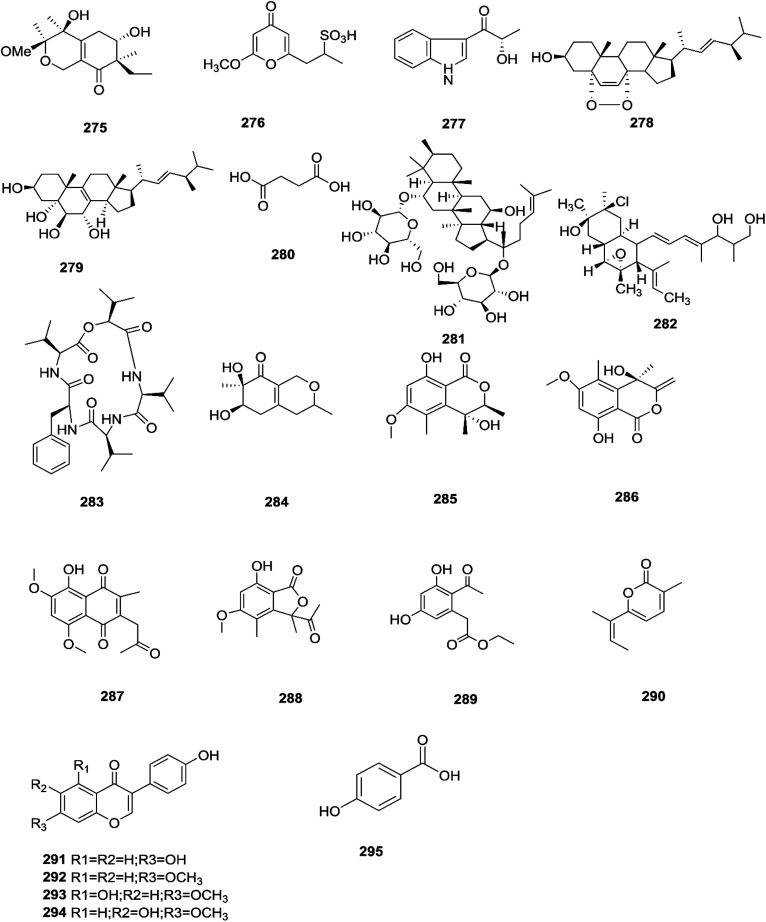
Various biologically active compounds (275–295) isolated from *Fusarium* sp.

**Fig. 24:**
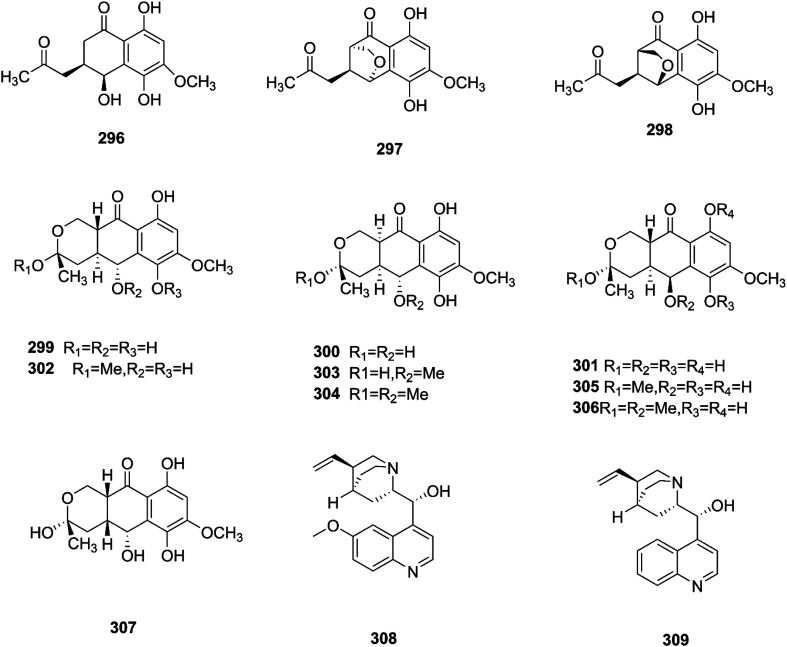
Various biologically active compounds (296–309) isolated from *Fusarium* sp.

Sansalvamide is a potentially promising anti-MCV agent as it is active against the virus-encoded type-1 topoisomerase, an enzyme likely to be required for MCV replication.^107^ Sansalvamide A was found to inhibit topoisomerase-catalyzed DNA relaxation; it also inhibited DNA binding and therefore covalent complex formation, but not resealing of a DNA nick in a preformed covalent complex, thereby specifying the part of the protein sensitive to sansalvamide A.^107^ Fusaraisochromenone (284), fusaraisochromanone (285), (*R*)-3,4-dihydro-4,8-dihydroxy-6-methoxy-4,5-dimethyl-3-methyleneisochromen-1-one (286), 8-*O*-methyljavanicin (287), 3-acetyl-7-hydroxy-5-methoxyl-3*H*-isobenzofuran-l-one (288), curvulin (289), fusalanipyrone (290), daidzein (291), formononetin (292), 7-*O*-methyl genistein (293), kakkatin (294), *p*-hydroxy benzoic acid (295) and tyrosol (23) were isolated from *Fusarium* sp. PDB51F5, which was reported to have cytotoxic activity against various cell lines including oral human carcinoma cells (KB), lung cancer cells (NCI-H137), human breast cancer cells (MCF-7) and non-cancerous Vero cells with IC_50_ ranging from 148 to 162 μM.^108^ Eleven new dihydronaphthalenones (296–306), together with five known compounds, were isolated from the endophytic fungus *Fusarium* sp. BCC14842, 4-hydroxydihydronorjavanicin (296), dihydronaphthalenone (297), and its diastereomer (298), 5-hydroxydihydrofusarubins A, B and D (299–301), and the methyl ether derivatives known as 5-hydroxy3-methoxydihydrofusarubin A (302), 5-methoxydihydrofusarubin B (303), 3,5-dimethoxydihydrofusarubin B (304), 5-hydroxy-3-methoxydihydrofusarubin D (305), 3,5-dimethoxydihydrofusarubin D (306), and 5-hydroxydihydrofusarubin C (307), javanicin (54), bostrycoidin (29), anhydrofusarubin (26), and 3-*O*-methylfusarubin (65).^109^ Compounds 298 and 302 exhibited weak to moderate anti-mycobacterial activity against *Mycobacterium tuberculosis* ranging from 25.0–50.0 μg mL^−1^, while compounds 297, 299, 327, 300, and 307 showed only cytotoxic activity against KB and NCI-H187 cell lines (IC_50_ values of 1.62–31.69 μg mL^−1^).^109^*Fusarium* species are capable of producing quinine (308) and cinchonidine (309) in synthetic liquid medium from different plant organs of *Cinchona calisaya*.^110^ Quinine has been used as the main medication for malaria disease due to its effectivity against the erythrocytic stage of the parasite *P. falciparum*.^110^ Cinchonidine has antimalarial activity, but it is not as effective as quinine.^111^ Quinine's antimalarial activity is due to its ability to inhibit the formation of hemozoin by forming five coordinate complexes with the porphyrin through their benzylic C-9 hydroxy groups, as well as an intramolecular hydrogen bond between the propionate side chain of Fe(iii) PPIX and the protonated quinuclidine nitrogen atom of the alkaloid. This complex inhibits the formation of hemozoin.^111^ This could justify further chemical investigation of these species for novel antimalarial drug discovery. The chemical structures 275–309 are shown in Fig. 23 and 24.

The above data demonstrate the outstanding biocontrol potential of members of the genus *Fusarium* and supports the continued investigation of these fungi as sources of potential biocontrol agents. This genus is a prolific source of bioactive secondary metabolites and can contribute in a spectacular way to improving human health. We have reviewed the ability of endophytic *Fusarium* species to produce many useful metabolites applicable in the pharmaceutical and agricultural industries, and explained the mechanism of action of some compounds. Most of the isolated metabolites from endophytic *Fusarium* species would make promising lead compounds for the development of anti-inflammatory, antibacterial, antifungal, antiviral, and cytotoxic agents (Table 1).

**Table 1:** List of isolated potent compounds of various *Fusarium* species

Compound	*Fusarium* species	Host plant	Bioactivity
Fusarithioamide A (16)	*F. chlamydosporium*	*Anvillea garcinii*	Cytotoxic (0.4–0.8 μM)
			Antibacterial (3.1–4.4 μM)
Fusarithioamide B (19)	*F. chlamydosporium*	*Anvillea garcinii*	Cytotoxic (0.09–0.21 μM)
			Antifungal (1.9 μM)
			Antibacterial (2.5–3.1 μM)
Cerevisterol (194)	*F. equiseti*	*Salicornia bigelovii*	Cytotoxic (32.4–37.56 μM)
Fusaripeptide A (224)	*Fusarium* sp.	*Mentha longifolia*	Cytotoxic (5.71 μM)
			Antifungal (0.11–0.14 μM)
Integracide H (240)	*Fusarium* sp.	*Mentha longifolia*	Cytotoxic (0.18–1.32 μM)
Integracide I (241)	*Fusarium* sp.	*Mentha longifolia*	Cytotoxic (0.18–1.32 μM)
4*H*-1-Benzopyran-4-one-2,3-dihydro-5-hydroxy-8-(hydroxylmethyl)-2-methyl (263)	*Fusarium* sp.	*Kandelia candel*	Cytotoxic (2.8–5.6 μM)
Aspergisocoumrin A (264)	*Fusarium* sp.	*Kandelia candel*	Cytotoxic (2.8–5.6 μM)
Taxol (24)	*F. solani*	*Taxus brevifolia*	Cytotoxic (2.5–7.5 μM)
Vitexin (25)	*F. solani*	*Cajanus cajan*	Cytotoxic (0.67–0.74 μM)
Rohitukine (82)	*F. proliferatum*	*D. binectariferum*	Cytotoxic (0.3–7.3 μM)
5α,8α-Epidioxyergosta-6,22-dien-3β-ol (278)	*Fusarium* sp.	*Paris polyphylla* var. *yunnanensis*	Antimicrobial (86.7–92.8 μM)
Fusolanone B (39)	*F. solani*	*Rhizophora apiculata*	Antibacterial (6.25 μM)
Epicyclonerodiol oxide (73)	*F. proliferatum*	Green Chinese onion	Antibacterial (12.5–25 μM)
Cyclonerodiol lactone (74)	*F. proliferatum*	Green Chinese onion	Antibacterial (12.5–25 μM)
5-*O*-Methylsolaniol (77)	*F. proliferatum*	Green Chinese onion	Antibacterial (12.5–25 μM)
5-*O*-Methyljavanicin (78)	*F. proliferatum*	Green Chinese onion	Antibacterial (12.5–25 μM)
Methyl ether fusarubin (79)	*F. proliferatum*	Green Chinese onion	Antibacterial (12.5–25 μM)
Anhydrojavanicin (80)	*F. proliferatum*	Green Chinese onion	Antibacterial (12.5–25 μM)
Amoenamide C (124)	*F. sambucinum*	*Nicotiana tabacum*	Antibacterial (1 μM)
Shikonin (172)	*F. tricinctum*	*Lithospermum officinale*	Antibacterial (0.30–6.25 μM)
			Anti-HCV activity (25 ng mL^−1^)
Helvolic acid methyl ester (237)	*Fusarium* sp.	*Ficus carica*	Antibacterial (3.13–6.25 μM)
			Antifungal (12.5 μM)
Helvolic acid (238)	*Fusarium* sp.	*Ficus carica*	Antibacterial (3.13–6.25 μM)
			Antifungal (12.5 μM)
Hydrohelvolic acid (239)	*Fusarium* sp.	*Ficus carica*	Antibacterial (3.13–6.25 μM)
			Antifungal (12.5 μM)
Sclerotiamide B (125)	*F. sambucinum*	*Nicotiana tabacum*	Potent insecticidal (70.2–70.5%)
Sclerotiamide (126)	*F. sambucinum*	*Nicotiana tabacum*	Potent insecticidal (70.2–70.5%)
Notoamide B (127)	*F. sambucinum*	*Nicotiana tabacum*	Potent insecticidal (70.2–70.5%)
Quinine (308) & Cinchonidine (309)	*Fusarium* sp.	*C. calisaya*	Anti-malarial

## 3. Conclusion and future perspectives

**Fig. 25:**
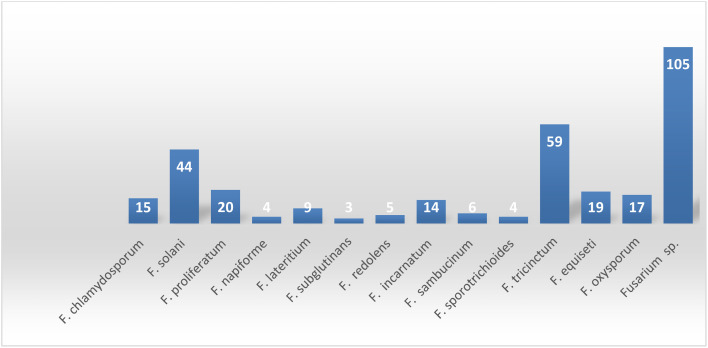
Number of secondary metabolites derived from different *Fusarium* species.

**Fig. 26:**
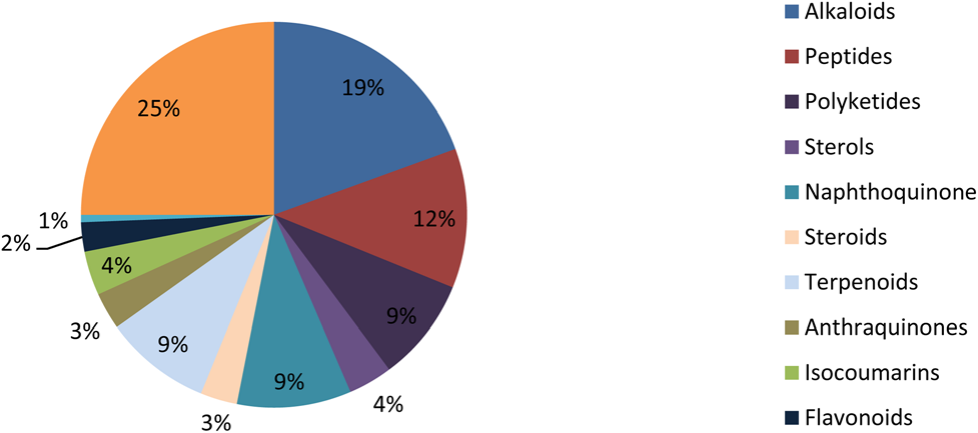
Secondary metabolite classes derived from the *Fusarium* genus.

**Fig. 27:**
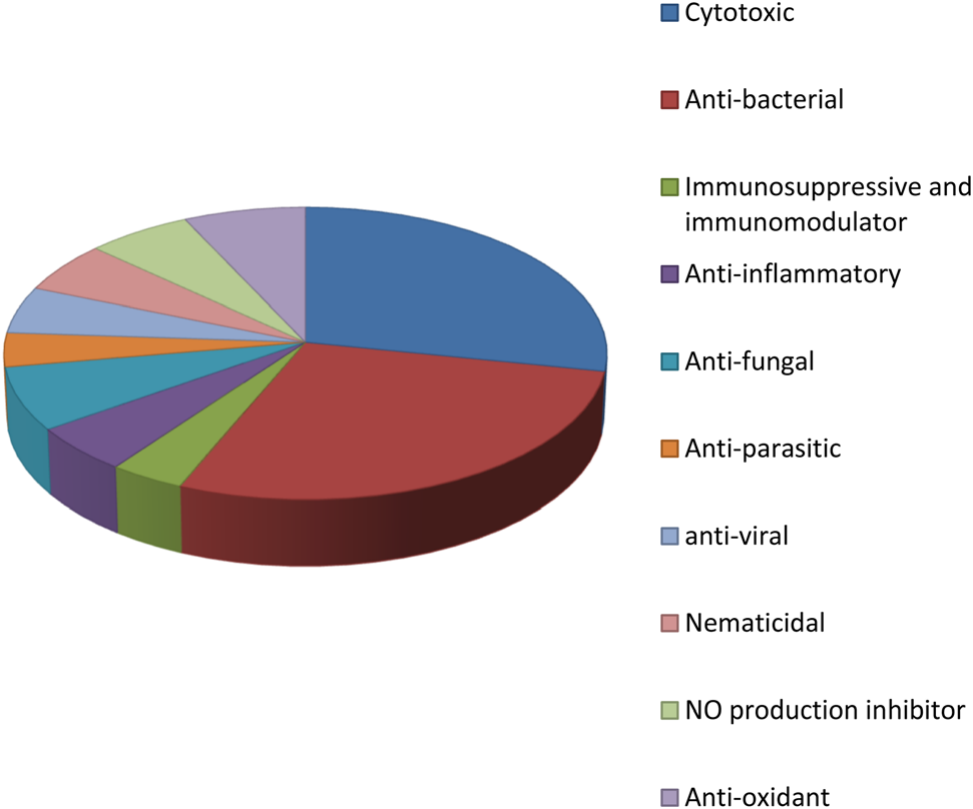
Bioactivities of natural products derived from the *Fusarium* genus.

**Fig. 28:**
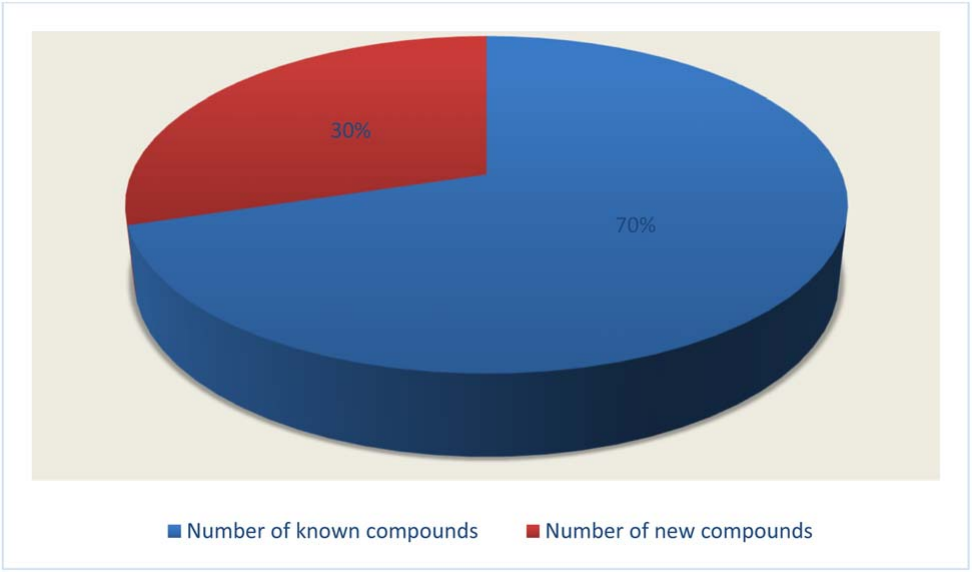
Number of new compounds that were firstly discovered from the endophytic *Fusarium* strains.

Fungal endophytes are the fungal population of the internal tissues of plants causing no apparent symptoms of disease. These endophytes are able to produce certain phyto-constituents originally attributed to their host plant, which is thought to be due to genetic recombination between the endophyte and the host plant that occurred in the evolutionary period. In addition, fungal endophytes are reported to produce new secondary metabolites, which may be totally different from those of the host plant, so they are a precious source of novel bioactive natural products and an alternative source for phytochemicals initially produced by higher plants. The *Fusarium* genus is among the well-known genera of fungal endophytes with many different species. *Fusarium* endophytes are considered to be a rich source of new secondary metabolites with valuable biological activities and a promising basis for drug discovery. Metagenomics studies revealed the potential for the discovery of new species within the genus *Fusarium* as there are several species that remain unidentified. This review reported the various phytoconstituents isolated from fourteen *Fusarium* species; *F. chlamydosporum*, *F. proliferatum*, *F. solani* and *F. oxysporum* are among the most isolated and identified endophytic *Fusarium* strains, see Fig. 25. The literature survey revealed that the varied abundance of secondary metabolites differs from year to year, for example only 15 compounds were reported from 1999 to 2010, while from 2011 to 2016, there was a significant increase in newly identified compounds, up to 112. The recent advances in chemical tools such as LC-MS could explain the increased discovery of new metabolites. *Fusarium* species have the ability to produce a varied array of secondary metabolites such as sterols, polyketides, alkaloids, terpenes, peptides and other compounds, see Fig. 26. Alkaloids are the most prolific chemical class produced by different *Fusarium* species followed by peptides and naphthoquinones, as illustrated in Fig. 26. Most of the isolated secondary metabolites showed diverse biological activities, which ensures that the curiosity in studying *Fusarium* endophytes is increased. Three hundred and nine compounds were reported, among them ninety two novel compounds, representing 23% of the isolated compounds, see Fig. 28. The isolated compounds exhibited multidimensional bioactivities, such as antimicrobial, antiviral, anticancer, antioxidative, anti-parasitic and immunomodulatory, see Fig. 27. Further investigations are recommended concerning endophytes to achieve promising scientific discoveries; these investigations should be directed toward fungal endophytes associated with plants of high medicinal value in order to produce the same phytochemicals as those isolated from the plants in an economical way. In addition, fungal endophytes can be manipulated genetically in order to produce high concentrations of certain natural products with high medicinal importance. Finally, intensive attention should be paid to the study of the mechanism of action of the frequently isolated fungal metabolites, such as alkaloids, peptides and naphthoquinones.

## Conflicts of interest

There are no conflicts to declare.

## Supplementary Material

## References

[cit1] Guo B., Wang Y., Sun X., Tang K. (2008). Appl. Biochem. Microbiol..

[cit2] El-hawary S. S., Moawad A. S., Bahr H. S., Abdelmohsen U. R., Mohammed R. (2020). RSC Adv..

[cit3] Strobel G., Daisy B. (2003). Microbiol. Mol. Biol. Rev..

[cit4] Nisa H., Kamili A. N., Nawchoo I. A., Shafi S., Shameem N., Bandh S. A. (2015). Microb. Pathog..

[cit5] Tawfik N., Abdo R., Abbott G., Abdelmohsen U. R., Edrada-Ebel R., Haggag E. (2017). J. Adv. Pharm. Educ. Res..

[cit6] Alhadrami H. A., Sayed A. M., El-Gendy A. O., Shamikh Y. I., Gaber Y., Bakeer W., Sheirf N. H., Attia E. Z., Shaban G. M., Khalifa B. A., Ngwa C. J., Pradel G., Rateb M. E., Hassan H. M., Alkhalifah D. H. M., Abdelmohsen U. R., Hozzein W. N. (2021). Sci. Rep..

[cit7] Mao Z., Zhang W., Wu C., Feng H., Peng Y., Shahid H., Cui Z., Ding P., Shan T. (2021). BMC Microbiol..

[cit8] Singh A., Kumar J., Sharma V. K., Singh D. K., Kumari P., Nishad J. H., Gautam V. S., Kharwar R. N. (2021). S. Afr. J. Bot..

[cit9] Veloz E., Portero C., Narvaez-Trujillo A. (2021). Adv. Microbiol..

[cit10] Yadav G., Meena M. (2021). Biotechnol. Rep..

[cit11] Nucci M., Anaissie E. (2007). Clin. Microbiol. Rev..

[cit12] Alhadrami H. A., Sayed A. M., El-Gendy A. O., Shamikh Y. I., Gaber Y., Bakeer W., Sheirf N. H., Attia E. Z., Shaban G. M., Khalifa B. A., Ngwa C. J., Pradel G., Rateb M. E., Hassan H. M., Alkhalifah D. H. M., Abdelmohsen U. R., Hozzein W. N. (2021). Sci. Rep..

[cit13] Stecher G., Tamura K., Kumar S. (2020). Mol. Biol. Evol..

[cit14] Wang Z. F., Zhang W., Xiao L., Zhou Y. M., Du F. Y. (2020). Nat. Prod. Res..

[cit15] Cai P., Smith D., Katz B., Pearce C., Venables D., Houck D. (1998). J. Nat. Prod..

[cit16] Al-Rabia M. W., Mohamed G. A., Ibrahim S. R. M., Asfour H. Z. (2021). Nat. Prod. Res..

[cit17] Al-Rabia M. W., Mohamed G. A., Ibrahim S. R. M., Asfour H. Z. (2021). Nat. Prod. Res..

[cit18] Ibrahim S. R. M., Mohamed G. A., Al Haidari R. A., Zayed M. F., El-Kholy A. A., Elkhayat E. S., Ross S. A. (2018). Bioorg. Med. Chem..

[cit19] Yadav T. C., Kumar N., Raj U., Goel N., Vardawaj P. K., Prasad R., Pruthi V. (2020). J. Biomol. Struct. Dyn..

[cit20] Kuriakose G. C., Arathi B. P., Divya Lakshmanan M., Jiby M. V., Gudde R. S., Jayabhaskaran C. (2020). Front. Oncol..

[cit21] Marupudi N. I., Han J. E., Li K. W., Renard V. M., Tyler B. M., Brem H. (2007). Expert Opin. Drug Saf..

[cit22] Tang P. J., Zhang Z. H., Niu L. L., Gu C. B., Zheng W. Y., Cui H. C., Yuan X. H. (2021). Biotechnol. Lett..

[cit23] He M., Min J. W., Kong W. L., He X. H., Li J. X., Peng B. W. (2016). Fitoterapia.

[cit24] Khan N., Afroz F., Begum M. N., Roy Rony S., Sharmin S., Moni F., Mahmood Hasan C., Shaha K., Sohrab M. H. (2018). Toxicol. Rep..

[cit25] Choi H. G., Song J. H., Park M., Kim S., Kim C. E., Kang K. S., Shim S. H. (2020). Biomolecules.

[cit26] Adorisio S., Fierabracci A., Muscari I., Liberati A. M., Cannarile L., Thuy T. T., Sung T. V., Sohrab H., Hasan C. M., Ayroldi E., Riccardi C., Mazid A., Delfino D. V. (2019). Toxins.

[cit27] Haraguchi H., Yokoyama K., Oike S., Ito M., Nozaki H. (1997). Arch. Microbiol..

[cit28] Zhou G., Qiao L., Zhang X., Sun C., Che Q., Zhang G., Zhu T., Gu Q., Li D. (2019). Phytochemistry.

[cit29] Mamur S., Ünal F., Yılmaz S., Erikel E., Yüzbaşıoğlu D. (2020). Drug Chem. Toxicol..

[cit30] Kyekyeku J. O., Kusari S., Adosraku R. K., Bullach A., Golz C., Strohmann C., Spiteller M. (2017). Fitoterapia.

[cit31] Chowdhury N. S., Sohrab M. H., Rana M. S., Hasan C. M., Jamshidi S., Rahman K. M. (2017). J. Nat. Prod..

[cit32] Shen W. Y., Bai R., Wang A. R., He J. Y., Wang H., Zhang Y., Zhao X. F., Dong J. Y. (2016). Nat. Prod. Res..

[cit33] Ran X., Zhang G., Li S., Wang J. (2017). Afr. Health Sci..

[cit34] Shweta S., Zuehlke S., Ramesha B. T., Priti V., Mohana Kumar P., Ravikanth G., Spiteller M., Vasudeva R., Uma Shaanker R. (2010). Phytochemistry.

[cit35] Hartmann J. T., Lipp H. P. (2006). Drug Saf..

[cit36] Shah A., Rather M. A., Hassan Q. P., Aga M. A., Mushtaq S., Shah A. M., Hussain A., Baba S. A., Ahmad Z. (2017). J. Appl. Microbiol..

[cit37] Dame Z. T., Silima B., Gryzenhout M., van Ree T. (2016). Nat. Prod. Res..

[cit38] Toghueo R. M. K. (2020). Mycology.

[cit39] Jiang C.-X., Li J., Zhang J.-M., Jin X.-J., Yu B., Fang J.-G., Wu Q.-X. (2019). J. Agric. Food Chem..

[cit40] Mohana Kumara P., Zuehlke S., Priti V., Ramesha B. T., Shweta S., Ravikanth G., Vasudeva R., Santhoshkumar T. R., Spiteller M., Uma Shaanker R. (2012). Antonie van Leeuwenhoek.

[cit41] Kumar V., Guru S. K., Jain S. K., Joshi P., Gandhi S. G., Bharate S. B., Bhushan S., Bharate S. S., Vishwakarma R. A. (2016). Bioorg. Med. Chem. Lett..

[cit42] Norred W. P., Bacon C. W., Riley R. T., Voss K. A., Meredith F. I. (1999). Mycopathologia.

[cit43] Zakaria L. (2017). Mycotoxins.

[cit44] Supratman U., Hirai N., Sato S., Watanabe K., Malik A., Annas S., Harneti D., Maharani R., Koseki T., Shiono Y. (2021). Nat. Prod. Res..

[cit45] Chutulo E. C., Chalannavar R. K., Pramod Kumar P. (2020). Stud. Fungi.

[cit46] Lee D., Choi H. G., Hwang J. H., Shim S. H., Kang K. S. (2020). Antioxidants.

[cit47] Zhong Z.-J., Lv Z.-C., Ouyang Y.-T., Hu Y.-X., Wang R., Peng Y.-H., Xu L.-X. (2022). J. Asian Nat. Prod. Res..

[cit48] Lin R., Kim H., Hong J., Li Q.-J. (2014). ACS Med. Chem. Lett..

[cit49] Park H., Christian L. S., Kim M. J., Li Q. J., Hong J. (2020). J. Med. Chem..

[cit50] Kim H., Baker J. B., Park Y., Park H. B., DeArmond P. D., Kim S. H., Fitzgerald M. C., Lee D. S., Hong J. (2010). Chem. - Asian J..

[cit51] Pan B. F., Su X., Hu B., Yang N., Chen Q., Wu W. (2015). Fitoterapia.

[cit52] Garyali S., Kumar A., Reddy M. S. (2013). J. Microbiol. Biotechnol..

[cit53] Nazir A., Hafeez S., Habeeb A. R. (2022). Arch. Microbiol..

[cit54] Wan E. R., Siew K., Heptinstall L., Walsh S. B. (2021). Clin. Kidney J..

[cit55] Xu L., Wang J., Zhao J., Li P., Shan T., Wang J., Li X., Zhou L. (2010). Nat. Prod. Commun..

[cit56] Gold R., Linker R. A., Stangel M. (2012). Clin. Immunol..

[cit57] Li L.-Y., Ding Y., Groth I., Menzel K.-D., Peschel G., Voigt K., Deng Z.-W., Sattler I., Lin W.-H. (2008). J. Asian Nat. Prod. Res..

[cit58] Ding L., Dahse H.-M., Hertweck C. (2012). J. Nat. Prod..

[cit59] Ding L., Peschel G., Hertweck C. (2012). Chembiochem.

[cit60] Ko Y. C., Choi H. S., Kim J. H., Kim S. L., Yun B. S., Lee D. S. (2020). Molecules.

[cit61] Zhang P., Yuan X.-L., Du Y.-M., Zhang H.-B., Shen G.-M., Zhang Z.-F., Liang Y.-J., Zhao D.-L., Xu K. (2019). J. Agric. Food Chem..

[cit62] Toghueo R. M. K. (2019). Mycology.

[cit63] Dai C., Xiao X., Sun F., Zhang Y., Hoyer D., Shen J., Tang S., Velkov T. (2019). Arch. Toxicol..

[cit64] Yang X., Liu P., Cui Y., Xiao B., Liu M., Song M., Huang W., Li Y. (2020). J. Agric. Food Chem..

[cit65] Bashyal B. P., Leslie Gunatilaka A. A. (2010). Nat. Prod. Res..

[cit66] Cao Y. M., Guo D. L., Jin M. Y., Tan L., Yang T. L., Deng F., Gu Y. C., Li X. H., Cao Z. X., Deng Y. (2021). Nat. Prod. Res..

[cit67] Moussa M., Ebrahim W., Bonus M., Gohlke H., Mándi A., Kurtán T., Hartmann R., Kalscheuer R., Lin W., Liu Z., Proksch P. (2019). RSC Adv..

[cit68] Ola A. R., Thomy D., Lai D., Brötz-Oesterhelt H., Proksch P. (2013). J. Nat. Prod..

[cit69] Sun W. J., Zhu H. T., Zhang T. Y., Zhang M. Y., Wang D., Yang C. R., Zhang Y. X., Zhang Y. J. (2018). Nat. Prod. Bioprospect..

[cit70] Hemphill C. F. P., Sureechatchaiyan P., Kassack M. U., Orfali R. S., Lin W., Daletos G., Proksch P. (2017). J. Antibiot..

[cit71] Zaher A. M., Makboul M. A., Moharram A. M., Tekwani B. L., Calderón A. I. (2015). J. Antibiot..

[cit72] Olleik H., Nicoletti C., Lafond M., Courvoisier-Dezord E., Xue P., Hijazi A., Baydoun E., Perrier J., Maresca M. (2019). Toxins.

[cit73] Zhang Y., Zhao J., Wang J., Shan T., Mou Y., Zhou L., Wang J. (2011). Nat. Prod. Commun..

[cit74] Mollaei S., Khanehbarndaz O., Gerami-Khashal Z., Ebadi M. (2019). Phytochemistry.

[cit75] Wang H., Liu Z., Li X., Zhao R., Pu Y., Wu H., Guan W. (2018). Exp. Ther. Med..

[cit76] Zhang J., Liu D., Wang H., Liu T., Xin Z. (2015). Eur. Food Res. Technol..

[cit77] Ma J. T., Du J. X., Zhang Y., Liu J. K., Feng T., He J. (2022). Fitoterapia.

[cit78] Zhao D.-L., Liu J., Han X.-B., Wang M., Peng Y.-L., Ma S.-Q., Cao F., Li Y.-Q., Zhang C.-S. (2022). Phytochemistry.

[cit79] Wang H., Liu T., Xin Z. (2014). Eur. Food Res. Technol..

[cit80] Alam M. B., Chowdhury N. S., Sohrab M. H., Rana M. S., Hasan C. M., Lee S. H. (2020). Biomolecules.

[cit81] Ben Rhouma M., Kriaa M., Ben Nasr Y., Mellouli L., Kammoun R. (2020). BioMed Res. Int..

[cit82] Wang Q. X., Li S. F., Zhao F., Dai H. Q., Bao L., Ding R., Gao H., Zhang L. X., Wen H. A., Liu H. W. (2011). Fitoterapia.

[cit83] Nascimento L., Conti R., Turatti, Cavalcanti B., Costa-Lotufo L., Pessoa C., Moraes M., de Toledo J., Cruz A., Cruz A. K., Pupo M. (2012). Rev. Bras. Farmacogn..

[cit84] Le T. T. M., Hoang A. T. H., Nguyen N. P., Le T. T. B., Trinh H. T. T., Vo T. T. B., Quyen D. V. (2020). Biotechnol. Lett..

[cit85] Damar U., Gersner R., Johnstone J. T., Schachter S., Rotenberg A. (2016). Expert Rev. Neurother..

[cit86] Gao H., Li G., Peng X. P., Lou H. X. (2020). Nat. Prod. Res..

[cit87] Xiao W.-J., Chen H.-Q., Wang H., Cai C.-H., Mei W.-L., Dai H.-F. (2018). Fitoterapia.

[cit88] Maharjan S., Lee S. B., Kim G. J., Cho S. J., Nam J. W., Chin J., Choi H. (2020). Molecules.

[cit89] Pan F., Hou K., Li D. D., Su T. J., Wu W. (2019). J. Biosci. Bioeng..

[cit90] Katoch M., Bindu K., Phull S., Verma M. K. (2017). Microbiology.

[cit91] Ibrahim S. R. M., Abdallah H. M., Elkhayat E. S., Al Musayeib N. M., Asfour H. Z., Zayed M. F., Mohamed G. A. (2018). J. Asian Nat. Prod. Res..

[cit92] Yang S.-X., Gao J.-M., Zhang Q., Laatsch H. (2011). Bioorg. Med. Chem. Lett..

[cit93] Chen Z. M., Dong W. B., Li Z. H., Feng T., Liu J. K. (2014). J. Asian Nat. Prod. Res..

[cit94] Liang X. A., Ma Y. M., Zhang H. C., Liu R. (2016). Nat. Prod. Res..

[cit95] Xiao J. H., Zhang Y., Liang G. Y., Liu R. M., Li X. G., Zhang L. T., Chen D. X., Zhong J. J. (2017). Exp. Biol. Med..

[cit96] Ibrahim S. R. M., Abdallah H. M., Mohamed G. A., Ross S. A. (2016). Fitoterapia.

[cit97] Yang S.-X., Wang H.-P., Gao J.-M., Zhang Q., Laatsch H., Kuang Y. (2012). Org. Biomol. Chem..

[cit98] Chen Y., Wang G., Yuan Y., Zou G., Yang W., Tan Q., Kang W., She Z. (2022). Front. Chem..

[cit99] Huang Z., Yang J., She Z., Lin Y. (2012). Nat. Prod. Res..

[cit100] Lv F., Daletos G., Lin W., Proksch P. (2015). Nat. Prod. Commun..

[cit101] Yang S. X., Gao J. M., Laatsch H., Tian J. M., Pescitelli G. (2012). Chirality.

[cit102] Zhang L., Liu Y., Deng Z., Guo Z., Chen J., Tu X., Zou K. (2013). Nat. Prod. Commun..

[cit103] Huang Y., Zhao J., Zhou L., Wang M., Wang J., Li X., Chen Q. (2009). Nat. Prod. Commun..

[cit104] Im D. S. (2020). Biomolecules.

[cit105] Brady S. F., Clardy J. (2000). J. Nat. Prod..

[cit106] Belofsky G. N., Jensen P. R., Fenical W. (1999). Tetrahedron Lett..

[cit107] Hwang Y., Rowley D., Rhodes D., Gertsch J., Fenical W., Bushman F. (1999). Mol. Pharmacol..

[cit108] Boonyaketgoson S., Trisuwan K., Bussaban B., Rukachaisirikul V., Phongpaichit S. (2015). Tetrahedron Lett..

[cit109] Kornsakulkarna J., Dolsophonb K., Boonyuen N., Boonruangprapaa T., Rachtaweea P., Prabpaib S., Kongsaereeb P., Thongpanchang C. (2011). Tetrahedron.

[cit110] Hidayat I., Radiastuti N., Rahayu G., Achmadi S., Okane I. (2016). Curr. Res. Environ. Appl. Mycol..

[cit111] Kingston D. G. I., Cassera M. B. (2022). Prog. Chem. Org. Nat. Prod..

